# The LabelHash algorithm for substructure matching

**DOI:** 10.1186/1471-2105-11-555

**Published:** 2010-11-11

**Authors:** Mark Moll, Drew H Bryant, Lydia E Kavraki

**Affiliations:** 1 Department of Computer Science, Rice University, Houston, TX 77005, USA; 2 Department of Bioengineering, Rice University, Houston, TX 77005, USA; 3 Structural and Comp. Biology and Molec. Biophysics, Baylor College of Medicine, Houston, TX 77005, USA

## Abstract

**Background:**

There is an increasing number of proteins with known structure but unknown function. Determining their function would have a significant impact on understanding diseases and designing new therapeutics. However, experimental protein function determination is expensive and very time-consuming. Computational methods can facilitate function determination by identifying proteins that have high structural and chemical similarity.

**Results:**

We present LabelHash, a novel algorithm for matching substructural motifs to large collections of protein structures. The algorithm consists of two phases. In the first phase the proteins are preprocessed in a fashion that allows for instant lookup of partial matches to any motif. In the second phase, partial matches for a given motif are expanded to complete matches. The general applicability of the algorithm is demonstrated with three different case studies. First, we show that we can accurately identify members of the enolase superfamily with a single motif. Next, we demonstrate how LabelHash can complement SOIPPA, an algorithm for motif identification and pairwise substructure alignment. Finally, a large collection of Catalytic Site Atlas motifs is used to benchmark the performance of the algorithm. LabelHash runs very efficiently in parallel; matching a motif against all proteins in the 95% sequence identity filtered non-redundant Protein Data Bank typically takes no more than a few minutes. The LabelHash algorithm is available through a web server and as a suite of standalone programs at http://labelhash.kavrakilab.org. The output of the LabelHash algorithm can be further analyzed with Chimera through a plugin that we developed for this purpose.

**Conclusions:**

LabelHash is an efficient, versatile algorithm for large-scale substructure matching. When LabelHash is running in parallel, motifs can typically be matched against the *entire *PDB on the order of minutes. The algorithm is able to identify functional homologs beyond the twilight zone of sequence identity and even beyond fold similarity. The three case studies presented in this paper illustrate the versatility of the algorithm.

## Background

High-throughput methods for structure determination have greatly increased the number of proteins with known structure in the Protein Data Bank (PDB) [1]. Structural genomics initiatives [2-4] have contributed not only to this increase in *number*, but have also increased the *diversity *of known protein structures. The function of most proteins is still poorly understood or even completely unknown. Automated functional annotation methods make it possible to fill in some of the gaps of missing information. Such methods can be a critical component of computational drug design and protein engineering. Their applicability, however, goes beyond traditional applications. For example, a nuanced and detailed understanding of protein function can also provide insight into the roles hub proteins play in protein interaction networks. Sequence-based methods are an established way for detecting functional similarity [5-8], but sequence similarity does not always imply functional similarity and vice versa. Structural analysis using the entire PDB allows for the discovery of similar function in proteins with very different sequences and even different folds [9]. For an overview of current approaches in sequence- and structure-based methods see [2,10,11].

Although it is possible to compare structures at the fold level [12,13], or by comparing pockets and clefts [14-16], this work focuses on substructure matching methods. Substructure matching methods aim to find a common substructure *motif *(sometimes also called a *template*) within one or more protein structures. Substructure matching methods can be used to identify functional similarity in cases where sequence similarity or fold similarity between homologous proteins is low (such as in cases of convergent evolution). The *identification *of an important substructure that forms a motif can be separated from the process of *matching *the motif against a number of structures. Several methods have been proposed to identify residues that are of functional importance [17-19]. Typically, such methods require as input a family of proteins that are known to be functionally similar. Once a structural motif has been identified that characterizes a given function or family, it is still a challenging problem to screen all the structures in the PDB for occurrences of substructures similar to this motif, and determine functional similarity. A wide variety of substructure matching methods have been proposed, such as: TESS [20], SPASM [21], CavBase [22], eF-site [23], ASSAM [24], PINTS [25], Jess [26], SuMo [27], SiteEngine [28], Query3 D [29], ProFunc [30], ProKnow [31], SitesBase [32], GIRAF [33], MASH [34], SOIPPA [35,36], FEATURE [37], and pevoSOAR [38]. These methods mainly differ in (1) the representation of structural motifs, (2) the motif matching algorithm, and (3) the statistics used to determine significance of match. Representations for substructural motifs include: C_α _coordinates with residue labels (with side-chain centroid [21] or without [25,34]), physicochemical pseudo-centers [28], graphs [22,24,35], general sets of constraints on atom positions and residue types [26], binding surfaces annotated with evolutionary conservation [38], or learnt vectors of features (such as the presence of a residue type of metal ion) occurring at certain distance ranges [37]. Such representations can then be matched using a variety of techniques such as: geometric hashing [20,28,32,39-41], depth-first search [21,25,29,34], graph algorithms (clique detection [22,23,27], subgraph isomorphism [24]), and constraint solvers [26]. To assess the statistical significance of matches the use of Extreme Value Distributions [36,42], mixtures of Gaussians [26], and a non-parametric model [35,43] have been proposed.

This paper describes a novel method for rapidly matching a motif against all known structures in the PDB (or any arbitrary subset thereof). It addresses several algorithmic and system design issues that allow it to be run in parallel and obtain near real-time performance. The method makes very few assumptions about the motif. For instance, a motif does not necessarily have to represent a cavity or binding site. The method was designed to be easy to use by both novice and expert users: the default parameters work in variety of scenarios, but can be easily changed to control the desired output. Through three case studies we demonstrate the versatility of the method and the ability to obtain highly sensitive and specific results.

## Results and Discussion

### The LabelHash Algorithm

We are interested in matching a structural motif against a large set of target structures. The structural motif is defined by the backbone C*_α _*coordinates of a number of residues and (optionally) allowed residue substitutions for each motif residue which are encoded as labels. Previous work has established that this is a feasible representation because it can find biologically relevant results [17,34,42,44].

The method presented below is called LabelHash. We will first give a high-level description. The method builds a hash table for *n*-tuples of residues that occur in a set of targets. In spirit LabelHash is reminiscent of the geometric hashing technique [40], but the particulars of the approach are very different. The *n*-tuples are hashed based on the residues' labels. Each *n*-tuple has to satisfy certain geometric constraints. The data in the hash table is indexed in a way that allows fast parallel access. Using this table we can look up partial matches of size *n *in constant time. These partial matches are augmented in parallel to full matches with an algorithm similar to MASH [34]. Compared to geometric hashing [40], our method significantly reduces storage requirements. Relative to MASH, we further improve the specificity. Furthermore, in the LabelHash algorithm it is no longer required to use importance ranking of residues to guide the matching (as was done in MASH). In our previous work, this ranking was obtained using Evolutionary Trace (ET) information [45]. The LabelHash algorithm was designed to improve the (already high) accuracy of MASH and push the envelope of matching with only very few geometric constraints. We want motifs to be as general as possible to allow for future extensions and to facilitate motif design through a variety of methods. Our simple-to-use and extensible LabelHash algorithm is extremely fast and can be a critical component of an exploratory process of iterative and near-interactive design and refinement of substructure templates. The algorithm consists of two stages: a preprocessing stage and a stage where a motif is matched against the preprocessed data.

#### Preprocessing Stage

The preprocessing stage has to be performed only once for a given set of targets; any motif can then be matched against the same preprocessed data. The targets can consist of single chains, but it is also possible to treat entire domains as a single target. This is useful for motifs that span multiple chains. During the preprocessing stage we aim to find possible candidate partial matches. This is done by finding all *n*-tuples of residues that satisfy certain geometric constraints. We will call these *n*-tuples *reference sets*. Typically, *n *is small; in our experiments we use 3-tuples. All valid reference sets for all targets are stored in a hash map, a data structure for *key/value *pairs that allows for constant time insertions and lookups (on average). In our case, each *key *is a sorted *n*-tuple of residue labels, and the *value *consists of a list of reference sets that contain residues with these labels in any order. So for any reference set in a motif we can instantly find all occurrences in all targets. Notice that in contrast to geometric hashing [40] we do not store transformed copies of the targets for each reference set, which allows us to store many more reference sets in the same amount of memory.

In our current implementation the geometric constraints apply to the C*_α _*coordinates of each residue, but there is no fundamental reason why other points such as C*_β_*'s or physicochemical pseudo-centers [22,46] cannot be used instead. We have defined the following four constraints on *valid *reference sets:

• Each C*_α _*in a reference set has to be within a distance *d*_maxmindist _from its nearest neighboring C*_α _*.

• The maximum distance between any two C*_α_*'s within a reference set is restricted to be less than *d*_diameter_.

• Each residue has to be within distance *d*_maxdepth _from the molecular surface. The distance is measured from the atom closest to the surface.

• At least one residue has to be within distance *d*_maxmindepth _from the surface.

The first pair of constraints requires points in valid reference sets to be within close proximity of each other, and the second pair requires them to be within close proximity of the surface. The distance parameters that define these constraints should be picked large enough to allow for at least one valid reference set for each motif that one is willing to consider, but small enough to restrict the number of seed matches in the targets. One would perhaps expect that the storage requirements would be prohibitively expensive, but--as described in the Implementation and Methods sections--the required storage is still very reasonable. The values for the four distance parameters described above were chosen empirically and kept fixed for all experiments (see *Methods*).

#### Matching Stage

For a given motif of size *m *(*m *≥ *n*), the LabelHash algorithm can look up all matches to a submotif of fixed size *n*, and expand each partial match to a complete match using a depth-first search. The partial match expansion is a variant of the match augmentation algorithm [34] that consists of the following steps. First, it solves the residue label correspondence between a motif reference set and the matching reference sets stored in the LabelHash table. (If more than one correspondence exists, all of them are considered.) Next, the match is augmented one residue at a time, each time updating the optimal alignment that minimizes the RMSD. If a partial match has an RMSD greater than some threshold *ε*, it is rejected. For a given motif point, we find all residues in a target that are within some threshold distance (after alignment). This threshold is for simplicity usually also set to *ε*. The threshold *ε *is set to be sufficiently large (7 Å in our experiments) so that no interesting matches are missed.

The algorithm recursively augments each partial match with the addition of each candidate target residue. The residues added to a match during match augmentation are *not *subject to the geometric constraints of reference sets. In other words, residues that are not part of a reference set are allowed to be further from each other and more deeply buried in the core. For small-size reference sets, the requirement that a motif contains at least one reference set is therefore only a very mild constraint. As we will see in the next section, our approach is still highly sensitive and specific.

For a given motif, we generate all the valid reference sets for that motif. Any of these reference sets can be used as a starting point for matching. However, those reference sets that have the smallest number of matching reference sets in the LabelHash table may be more indicative of a unique function. Reference sets with a large number of matches are more likely to be common structural elements or due to chance. We could exhaustively try all possible reference sets, but for efficiency reasons we only process a fixed number of least common reference sets. Note that the selection of reference sets as seed matches is based only on frequency. In contrast, in our previous work, only one seed match was selected based on importance ordering frequently based on evolutionary importance [34]. Because of the preprocessing stage it now becomes feasible to expand matches from many different reference sets. The information stored inside a LabelHash file is stored so that only the relevant parts of the file need to be read from disk during matching.

The matching algorithm is flexible enough to give users full control over the kind of matches that are returned. It is possible to keep multiple matches per target or partial matches that match at least a certain minimum number of residues. The latter option can be useful for larger motifs where the functional significance of each motif point is not certain. In such a case, a 0.5 Å RMSD partial match of, say, 9 residues, might be preferable over a 2 Å complete match of 10 residues. With partial matches, the matches can be ranked by a scoring function that balances the importance of RMSD and the number of residues matched. One can also choose between keeping only the lowest RMSD match per target or all matches for a target, which may be desirable if the top-ranked matches for targets have very similar RMSD's. Finally, the number of motif reference sets that the algorithm uses for match augmentation can also be varied. Usually most matches are found with the first couple reference sets, but occasionally a larger number of reference sets need to be tried before the smallest RMSD match for each target is found. With the default settings (used for all results in this paper), the number is set to a conservative threshold of 15.

#### Statistical Significance of Matches

There is no universal RMSD cut-off that can be used to decide whether a match is significant, and picking a cut-off for a given motif and corresponding protein family is non-trivial. The cut-off depends on the structural variation within a protein family and the likelihood that a match to a non-homologous protein occurs due to chance. In our work we use a nonparametric model to compute the statistical significance of each match. This model is briefly summarized below and described in more detail in [43,47]. The model assumes that the matching algorithm returns for each target only the lowest RMSD, complete match to a motif. Keeping partial matches or multiple matches per target complicates the determination of the statistical significance of each match. This is an issue we plan to investigate in future work.

**Figure 1:**
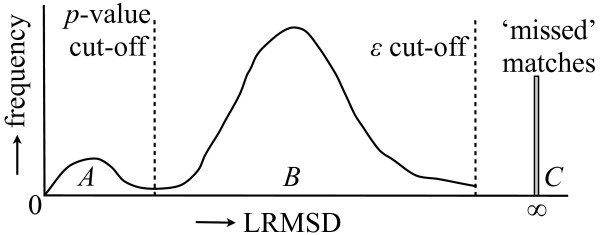
**Non-parametric model of statistical significance of matches**. Accounting for the 'missed' matches due to the RMSD cut-off parameter *ε *with a point-weight at ∞ allows for accurate estimation of the *p*-value of matches.

For a set of matches we can compute a probability density function over RMSD by smoothing the RMSD distribution using the Sheather-Jones optimal bandwidth [48]. An example distribution is shown in Figure 1. In an ideal case, functionally homologous targets would have a low RMSD and would be well-separated from the non-homologous targets. From this RMSD distribution, which we will call a *motif profile*, we can assign a *p*-value to a match by dividing the area under the curve to the left of the match's RMSD by the total area under the curve. For instance, for the match at the cut-off in Figure 1, the *p*-value would be $\frac{A}{A+B}$, where *A *and *B *are the areas under the curve to the left and right of the dotted line, respectively. However, the value of the distance cut-off parameter *ε *introduces an algorithmic bias that affects the computation of the statistical significance of a match. Other matches could be found if the value of *ε *were increased. To correct for this bias, we model the existence of these 'missed' matches by placing a point-weight proportional to their relative frequency at infinity. The *corrected p*-value is then $\frac{A}{A+B+C}$, where *C *is the weight of the missed matches. Finding missed matches is straightforward: for each target where no match was found, we simply check whether there are enough residues of the right types so that a match is possible. The residue frequencies are pre-computed for each target and stored inside the LabelHash tables.

It can be shown that for a motif of *n *residues our statistical model computes the *exact p*-value of matches with RMSD less than $\varepsilon /\sqrt{n}$, i.e., their *p*-value would not change if no *ε *threshold was used [43]. For example, for a 6-residue motif and *ε *= 7 Å, the *p*-values of all matches within 2.9 Å of the motif are exact.

### Implementation

#### Data Layout

We aimed for LabelHash to be scalable to all available structures in the Protein Data Bank, even as it continues to grow. The structural information, reference sets and indexing information require significant storage. The data layout is determined not only by the content, but also by the expected access patterns. Space-efficient storage of all data that supports computationally efficient access can contribute significantly to the overall speed of the algorithm. Typically, only a very small fraction of the data is accessed in matching a motif for two reasons. First, only a small number of reference sets in a motif is used for match expansion. Second, often we are only interested in matching a motif against a subset of all known structures (such as the non-redundant PDB or a given class/family of proteins). The data format should also be extensible (so that we can store additional attributes for each target in the future) and allow concurrent access to facilitate parallelized matching (see next subsection). In our current implementation we have chosen to use the Hierarchical Data Format (HDF5) [49], a standard file format used for large data sets. Conceptually, the HDF5 software library creates a file system within a file. With HDF5 one can easily create and change hierarchical groups of data sets, without having to worry about keeping all the indexing information up-to-date. It also allows for sophisticated compression schemes (which can be enabled per data set) and includes support for parallel I/O (by providing a software layer on top of MPI-IO).

The data is laid out as follows. For each target there is a group that contains data sets with the target's structural information, residue types, and any other information specific to the target that we may add in the future. For every possible set of *n *residue labels, we store all the reference sets with those labels in a large matrix with *n *columns, where *n *is the reference set size. Associated with each matrix is some additional indexing information that keeps track of which block of rows in each matrix contains indices into which target structure. The *n*-tuples take up the bulk of the data that needs to be stored, but, luckily, they are also very compressible. We chose to use SZIP [50] compression because of its high compression ratio and fast decompression speed. The LabelHash HDF5 file for the *entire *PDB (based on a snapshot from March 25, 2009), including reference sets and all metadata takes up 65 GB. Although this is a very large file, it still fits easily on commodity hard drives (and even some solid state drives), and a significant portion of the file could be kept in memory (e.g., in page cache) on a dedicated server.

#### Large-Scale Matching

Multi-core processors and distributed computing clusters are increasingly commonplace, and naturally we would like to take advantage of that. Both the preprocessing stage and the matching stage are parallelized, and a near-linear speed-up with the number of CPU cores can be achieved. In the preprocessing phase a master node asynchronously sends PDB id's to slave nodes, which read the corresponding PDB file, compute the reference sets, and send all data back to the master. The master node writes all data to disk. Although this seems suboptimal in terms of communication, it avoids the difficulties associated with parallel write access (if all nodes write to a single file) or a sequential merge of several files (if nodes write to separate files).

Matching in LabelHash is also easily parallelized. The targets to be matched are evenly divided over the nodes and each node matches a given motif against its targets independently. Once matching is finished, the match results are aggregated into one output file by one of the cores. This parallelization scheme could lead to load imbalance if matching against some targets takes significantly longer than others. In our experiments the number of targets was usually large enough and arbitrarily distributed over the nodes such that there was no imbalance. If load imbalance were to become an issue, it would be relatively easy to implement schemes that dynamically assign batches of targets to the nodes. Another potential performance bottleneck is the simultaneous disk access by the nodes. If all nodes independently try to read data from the LabelHash table, they can spend a significant amount time in system calls, waiting for disk access or seeking the right disk sectors. The bulk of the data that needs to be read consists of the *n*-tuples for the selected targets. This data is read synchronously using the HDF5-provided software layer on top of MPI-IO. The amount of data to be read by each node is roughly equal, so that idle time is minimized. Once the *n*-tuples have been read, the match augmentation phase starts. For each of its targets, a node will load the structural information from the LabelHash table and compute the best match(es). The computation time tends to be significantly larger than the disk access time, so that the nodes can read the data asynchronously with only minimal disk contention.

#### Algorithmic Issues

In the original implementation of match augmentation [34], the runtime was dominated by computing (a) RMSD alignments and (b) nearest neighbors for a motif point in a target structure. In addition to the performance improvements obtained through the pre-computation of reference sets, the LabelHash algorithm uses a fast, new algorithm and pre-computation, respectively, to speed up these two components.

The match augmentation algorithm iteratively updates the RMSD alignment with each residue added to a partial match. Since match augmentation is started from many matching reference sets and for each partial match there can be many possibilities for match augmentation, the computation of RMSD alignments can potentially be a performance bottleneck. Computing such alignments typically involves computing a covariance matrix and the largest eigenvalue/eigenvector pair of this matrix [51,52]. For small substructures the computational cost is dominated by the eigenvalue/eigenvector calculation. We use a recently proposed method to compute the optimal alignment by finding the largest root of the characteristic polynomial, instead of computing a matrix decomposition of the covariance matrix [53,54]. Using this method in our implementation, the time needed for RMSD calculations is drastically reduced to less than one tenth of the traditional approach.

To expand a partial match by one residue, the match augmentation algorithm looks for matching residues for the next motif point under the current optimal alignment. The matching residues are found using a proximity data structure. Several such data structures exist; in our implementation we use Geometric Near-neighbor Access Trees [55]. The key observation to speeding up proximity queries is that the nearest neighbors of a motif point are very similar to the nearest neighbors of the point in the target closest to this motif point. Suppose we are interested in all nearest neighbors within 7 Å of a motif point and the closest point in the target is *x *Å away. Then the set of nearest neighbors of this point that are within (7 + *x*) Å includes all the nearest neighbors of the motif point. Those within (7 - *x*) Å are guaranteed to be also nearest neighbors of the motif point, and only those within the (7 - *x*) Å to (7 + *x*) Å range need to be checked. So the nearest neighbors of an *arbitrary *point can simply be found by computing *one *nearest neighbor, and looking up the nearest neighbors of this neighbor. To be able to look up all nearest neighbors for an arbitrary point within a radius of *r *Å, we need to precompute nearest neighbors for each target within a radius of 2*r *Å, since *x *can be at most *r *Å. This is exactly what is currently implemented; the indices of the nearest neighbors and corresponding distances are stored (in compressed format) for each target point. This adds only marginally to the total table size, while providing significant speedups.

#### The LabelHash Server and Chimera Interface

The LabelHash algorithm has been made accessible through a web interface at http://labelhash.kavrakilab.org. A user can specify a motif by a PDB ID and a number of residue ID's. For each motif residue the user can optionally specify a number of alternate labels. The user can match against either the full PDB or the 95% sequence identity filtered non-redundant PDB (nrPDB_95_). Once the matches for a motif have been computed, an email is sent to the user which includes a URL for a web page with the match results. This page lists information for the top matches, including: the matched residues, the RMSD, the *p*-value, Enzyme Commission (EC) numbers and Gene Ontology (GO) annotations (if applicable), and a graphical rendering of the match aligned with the motif.

**Figure 2:**
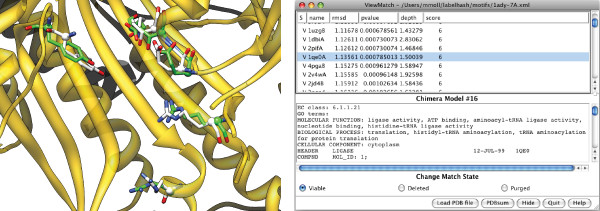
**Match visualization using Chimera**. **Left: **A match (in green) shown superimposed with a motif (in white), while the rest of the matching protein is shown in ribbon representation. **Right: **The ViewMatch controller window.

From the results page, one can also download an XML file that contains *all *the matches found. This match file can then be loaded in Chimera, a popular molecular visualization and analysis program [56]. For this, we have developed a plugin called ViewMatch. (It is derived from the Chimera ViewDock extension for visualizing results from the DOCK program.) The ViewMatch plugin allows the user to scroll through the list of matches. Figure 2 shows the user interface. In the main window a selected match is shown superimposed with the motif. Recall that, although all atoms in the matched residues are shown, only the C*_α _*atoms were used to compute the alignment. The C*_α _*atoms are shown as spheres. In the controller window on the right, all matches are listed in the top half with their RMSD to the motif, *p*-value and other attributes. By specifying constraints on the match attributes, the user can restrict the matches that are shown. The bottom half of the window shows additional information for the selected match, such as the EC classification and GO terms. By clicking the PDBsum button, the PDBsum web pages [57] are shown for the selected matches. This gives the user an enormous amount of information about a match.

We expect that advanced users may want to have more control over the matching parameters and the creation of LabelHash tables. For that reason, we have made available for download on the LabelHash web site a suite of command line tools (for Linux and OS X) to do just that. It includes programs to run LabelHash in parallel on a cluster of machines, as well as a Python interface.

### Testing

The LabelHash algorithm has been evaluated for speed, scalability, and functional annotation precision through a carefully selected set of case studies that demonstrate orthogonal features of the algorithm. First, the ability of LabelHash to identify common substructures at the superfamily level of SCOP [58] classification is validated within the Enolase Superfamily (ES). Second, the ability of LabelHash to construct motifs from the output of state-of-the-art methods and then quickly match them against the entire PDB is demonstrated with the output of SOIPPA [35], a motif discovery and structural alignment algorithm. Third, as a comprehensive functional annotation benchmark, LabelHash motifs are created from catalytic residues documented by the Catalytic Site Atlas (CSA) [59] and then matched against the nrPDB_95 _to assess per-motif functional annotation sensitivity and specificity. Finally, we evaluate the speed and scalability of the LabelHash algorithm by matching all motifs used in this paper against the nrPDB_95_. Together, these tests demonstrate the versatility and generality of LabelHash for a variety of functional annotation problems.

#### Identifying Members of the Enolase Superfamily

To demonstrate the ability of LabelHash to successfully identify motifs at the superfamily scale, spanning multiple EC classes, LabelHash was used to identify shared catalytic substructures among Enolase Superfamily (ES) [60] proteins. Previous work by Meng et al. [61] used the SPASM [62] substructure comparison method to investigate a conserved substructure within ES proteins. As a challenging validation experiment, LabelHash was used to identify the conserved substructure (ESmotif) among the same benchmark set of ES structures (ESdb) that were both defined previously by Meng et al. [61]. LabelHash was able to identify those structures included in the ESdb with high sensitivity and specificity. In addition to those structures included in the ESdb, matches were identified by LabelHash to additional ES proteins which have more recently been identified and to several proteins with only putative ES-like function.

**Figure 3:**
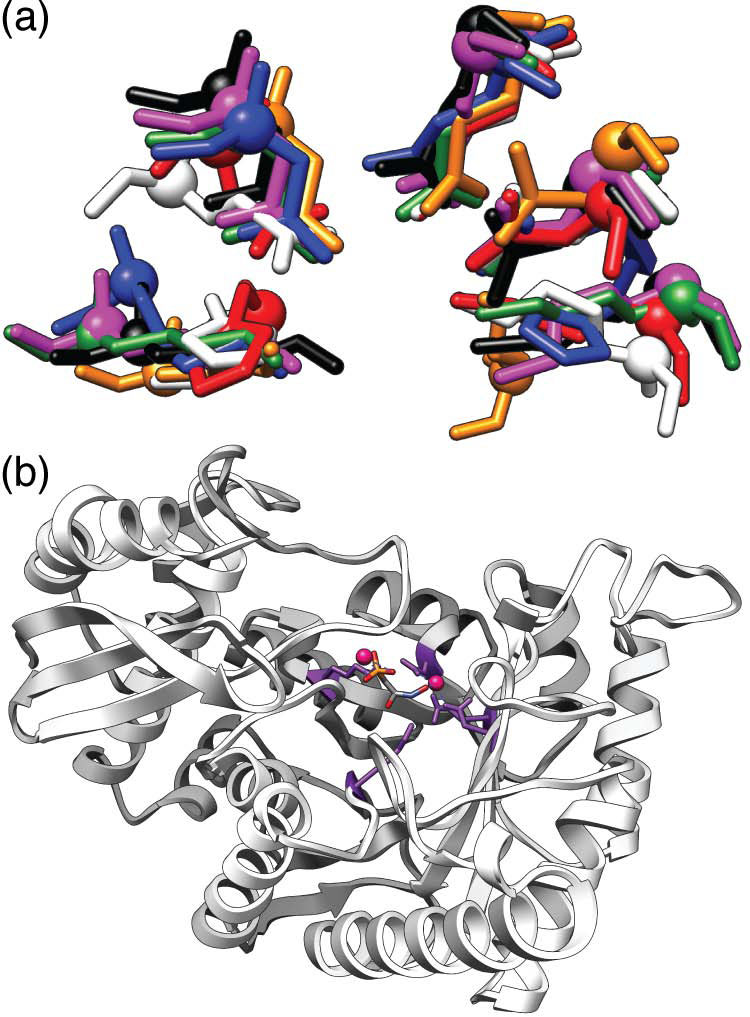
**The enolase superfamily (ES) motif**. **(a) **The substructure responsible for the conserved partial reaction among members of the ES consists of the five residues shown above from which the ES motif used here is derived [61]. **(b) **Enolase from *Saccharomyces cerevisiae *[PDB:1ELS] with demonstrated divalent ion coordination to the conserved superfamily substructure [68]. A single Mn^2+ ^is coordinated by a carboxylate triad (Asp, Glu, Asp in this enzyme) and a second Mn^2+ ^binds in a substrate specific manner [68].

The ES currently includes 7 major sub-groups spanning 20 families and 14 well-defined EC classes as defined by the Structure-Function Linkage Database (SFLD) [63]. As shown in Figure 3, a core of five residues directly mediates the conserved partial reaction among ES members. Modeling this substructure as a five-residue motif as done by Meng et al. [61] results in the following superfamily-specific motif (ES-motif): 164^KH^, 195^D^, 221^E^, 247^EDN^, 297^HK^; residues are numbered with respect to mandelate racemase structure [PDB:2MNR]. A benchmark set of ES structures defined by Meng et al. [61] (ESdb) was used as a positive test set; the nrPDB_95 _was used as a background structure set which also includes several overlapping structures belonging to the ES. Residues making up the conserved substructure among ES members are known to vary widely in C*_α _*RMSD (up to 2.3 Å C*_α _*RMSD) although the side-chains of the residues superimpose closely (as shown in Figure 3). By using LabelHash to enumerate all reasonable substructure matches based on C*_α _*RMSD and then filtering the resulting potential matches by side-chain RMSD, LabelHash successfully identifies ES members with high sensitivity and specificity using the approach outlined below.

To identify matches based on a different distance metric rather than C*_α _*deviation involves adding only a simple post-processing step to the initial substructure matches identified by LabelHash. For each target structure in the ESdb, many possible matches are first identified based on amino acid label compatibility and C*_α _*distance cutoffs to the ES motif. From these possible matches, a single "best" match is selected per target based upon minimum side-chain centroid RMSD to the motif rather than C*_α _*RMSD as done in previous experiments. The distribution of matches yielded by this process differs from the minimum C*_α _*distance distribution. For example, enumerating all possible matches in enolase structure [PDB:1EBH] results in 84 possible matches and from these possible matches, a "best" match can be selected based on *any *user-defined criteria. For ES we used side-chain centroid RMSD, but in general C*_α _*RMSD, surface accessibility, distance to a known ligand, or even colocation with a pocket/cavity could be used instead. For the ES, side-chain centroid RMSD is preferred over full-atom side-chain RMSD due to the fact that several different types of amino acids are possible at each position and defining an appropriate one-to-one atom mapping between side-chains of different amino acids is difficult to define. However, C*_β _*alignment is a viable alternative to the side-chain centroid if a pseudo atom is defined for glycine.

**Figure 4:**
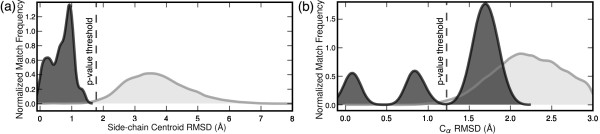
**LabelHash results for ES motif based on **[PDB:2MNR]. Dark gray denotes matches to structures in the ESdb as defined by Meng et al. [61] while light gray denotes matches to structures in the nrPDB_95_. The dashed line in each plot corresponds to the *p*-value threshold at *α *= 0.01. **(a) **The clear separation of the distributions of ESdb and nrPDB_95 _matches identified using minimum side-chain RMSD illustrates the high-specificity of the 2MNR-based motif. **(b) **Examining the distributions of matches based upon C*_α _*RMSD demonstrates the inseparability of ESdb matches from the majority of nrPDB_95 _structures if minimum C*_α _*RMSD is used alone for match selection.

Comparing the prediction performance of C*_α _*versus side-chain centroid RMSD for ES, as shown in Figure 4, demonstrates that alternative match selection measures, such as side-chain deviation, outperforms C*_α _*deviation for the ES. Using minimum C*_α _*RMSD as a match selection criteria per target, only 31% of structures in the ESdb are matched with statistical significance (*p*-value threshold of *α *= 0.01, 29 false negative matches). However, with minimum side-chain centroid RMSD as a match selection criterion per target, LabelHash achieves 95% sensitivity. The match RMSD distributions in Figure 4 reveal the higher discriminating power of side-chain centroid RMSD for the ES. Using side-chain centroid RMSD, the distribution of matches for the ESdb structures is easily separable from the much larger number of matches to nrPDB_95 _structures while C*_α _*RMSD alone results in the majority of ESdb matches to overlap with unrelated matches to structures in the nrPDB_95_.

Many matches to nrPDB_95 _structures still fall below the statistical significance threshold (as shown in Additional File 1) and these possibly false positive (FP) matches were further investigated. Out of the 47 total FP matches, 35 matches actually corresponded to structures that have now been identified to belong to ES as documented by the SFLD [63]. An additional 10 FP matches corresponded to structures that have only putatively defined functions, but may be related to the ES: [PDB:2QGY], [PDB:3CK5], [PDB:2OO6], [PDB:2PPG], [PDB:2OQH], [PDB:3CYJ], [PDB:1WUF], [PDB:1WUE], [PDB:2OZ8], and [PDB:2POZ]. The final 2 matches correspond to an aminoacylase from the M20 family [PDB:1YSJ] and human arginase I [PDB:2AEB]; both matches correspond to dimetal binding sites within the two different enzymes which bind Mn^2+ ^and Ni^2+^, respectively. Altogether, these results highlight the potential of LabelHash to identify similarities between remote homologs.

#### Combining LabelHash and SOIPPA

Recent work by Xie and Bourne on Sequence Order-Independent Profile-Profile Alignment (SOIPPA) [35] and SMAP [36] allows the alignment and identification of structurally similar, but sequentially distinct, sequence motifs. To further validate the LabelHash method, the alignment output of SOIPPA was used to create LabelHash motifs that are demonstrated to maintain the high-specificity of SOIPPA. LabelHash provides the means to search the entire nrPDB_95 _for matches to SOIPPA-derived motifs in a matter of minutes. These experiments illustrate how LabelHash can be used to further enhance available structural analysis methods by converting identified residues of interest directly to LabelHash motifs that can be efficiently scanned against large structural databases.

**Figure 5:**
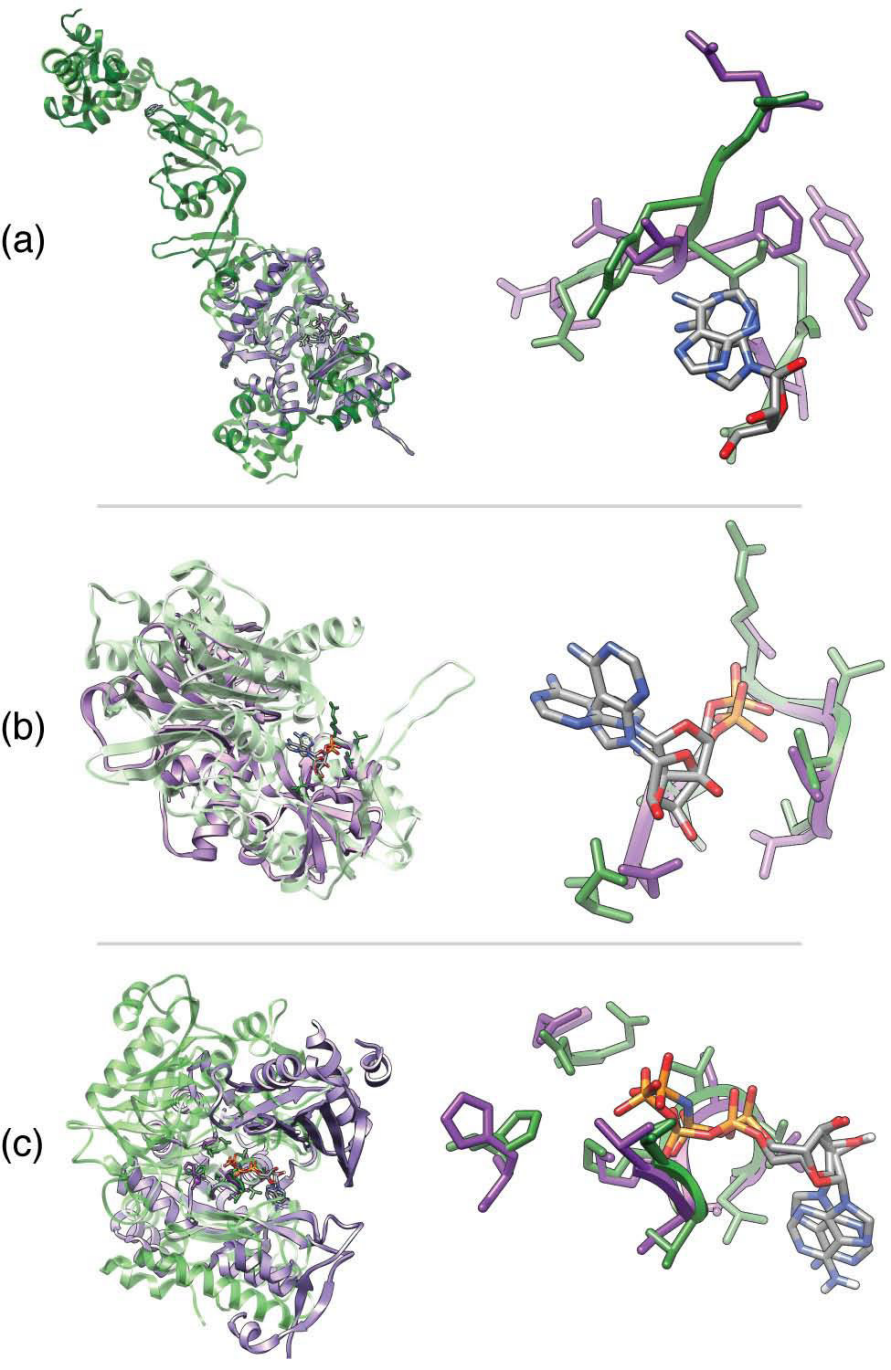
**SOIPPA motifs**. In each image, the motif substructure is colored green while the matched substructure is colored purple. All ligands neighboring either the motif or matched substructure are shown colored by atom type for reference; the ligand atoms are not used at any point during the LabelHash matching process. **(a) **LabelHash motif based on [PDB:1HQC] in C*_α _*RMSD alignment to the matched substructure from [PDB:1ZTF]; 8 matched residues. **(b) **LabelHash motif based on [PDB:1ECJ] in C*_α _*RMSD alignment to the matched substructure from [PDB:1H3D]; 7 matched residues. **(c) **LabelHash motif based on [PDB:1AYL] in C*_α _*RMSD alignment to the matched substructure from [PDB:1P9W]; 11 matched residues.

**Figure 6:**
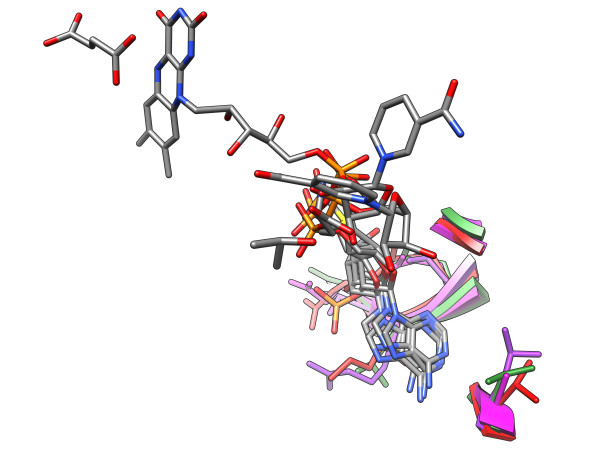
**SOIPPA-derived motif**. LabelHash motif constructed by combining multiple pairwise SOIPPA alignments in order to identify a common set of residues shared amongst all SOIPPA-aligned structures. Each of the four substructures used to construct the LabelHash motif are shown in red, green, purple, and fuchsia; the identified common set of 5 residues is shown for each substructure. The ligands neighboring each substructure are shown for reference and colored by atom type.

Several of the aligned sequence motifs identified by SOIPPA were used to construct the LabelHash motifs listed in Table 1. For each template structure in this table, a LabelHash motif was defined using the residues and alternate amino acid labels identified by SOIPPA alignment [35]. Specifically, to construct a LabelHash motif from the SOIPPA alignment output data, the coordinates of the SOIPPA-identified motif residues from *only *the template structure are combined with the set of alternate amino acid labels identified by SOIPPA-alignment; the coordinates of the structures aligned by SOIPPA (i.e., structures other than the template structure) are not used. Each LabelHash motif was then matched against the corresponding SOIPPA alignment structures in the table, in addition to the nrPDB_95 _as a background structure set, in order to assess the statistical significance of matches in each corresponding alignment structure. In all cases the correct match was identified with extremely low *p*-values. Both the LabelHash motif substructure and corresponding match substructures can be seen in Figures 5 and 6.

**Table 1:** SOIPPA-derived Motifs

Template Structure	SOIPPA-derived LabelHash Motif	SOIPPA-aligned Structure(s)	*p***-value of LabelHash-computed match**
1HQC	9^E^, 10^YI^, 11^IF^, 12^GE^, 13^Q^, 169^LV^, 171^QY^, 172^GA^	1ZTF	< 0.0009
1ECJ	367^D^, 369^IV^, 371^RT^, 372^G^, 373^TA^, 374^T^, 375^SL^	1H3D	< 0.0005
1AYL	232^H^, 250^ST^, 251^G^, 252^TS^, 253^GA^, 254^K^, 255^TS^, 256^T^, 257^LT^, 268^DG^, 269^DE^	1P9W	< 0.0006
1ZQ9	29^G^, 50^ET^, 51^LRKS^, 52^DTEQ^, 79^VLA^	1CYD, 1D4D, 1UWK	< 0.0005

Combining multiple pairwise SOIPPA alignments allows for the construction of a single LabelHash motif that comprises the common SOIPPA motif residues shared between each pairwise alignment. For example, based upon SOIPPA pairwise alignments between SAM-dependent methyltransferase [PDB:1ZQ9] and each of urocanase [PDB:1UWK], carbonyl reductase [PDB:1CYD], and flavocytochrome c3 fumarate [PDB:1D4D], a single LabelHash motif was derived using the subset of residues from [PDB:1ZQ9] identified by SOIPPA [35] within each of the three aforementioned pairwise alignments and the alternative amino acid labels for residues in each pairwise SOIPPA alignment. The resulting LabelHash motif correctly identifies the corresponding residues in each of [PDB:1UWK], [PDB:1CYD], and [PDB:1D4D] without LabelHash taking into account any knowledge of each of these three target structures beyond the alternate labels required. The [PDB:1ZQ9]-based LabelHash motif was then matched against the full PDB to search for other structures with statistically significant similarity to the shared substructure modeled by the motif.

**Figure 7:**
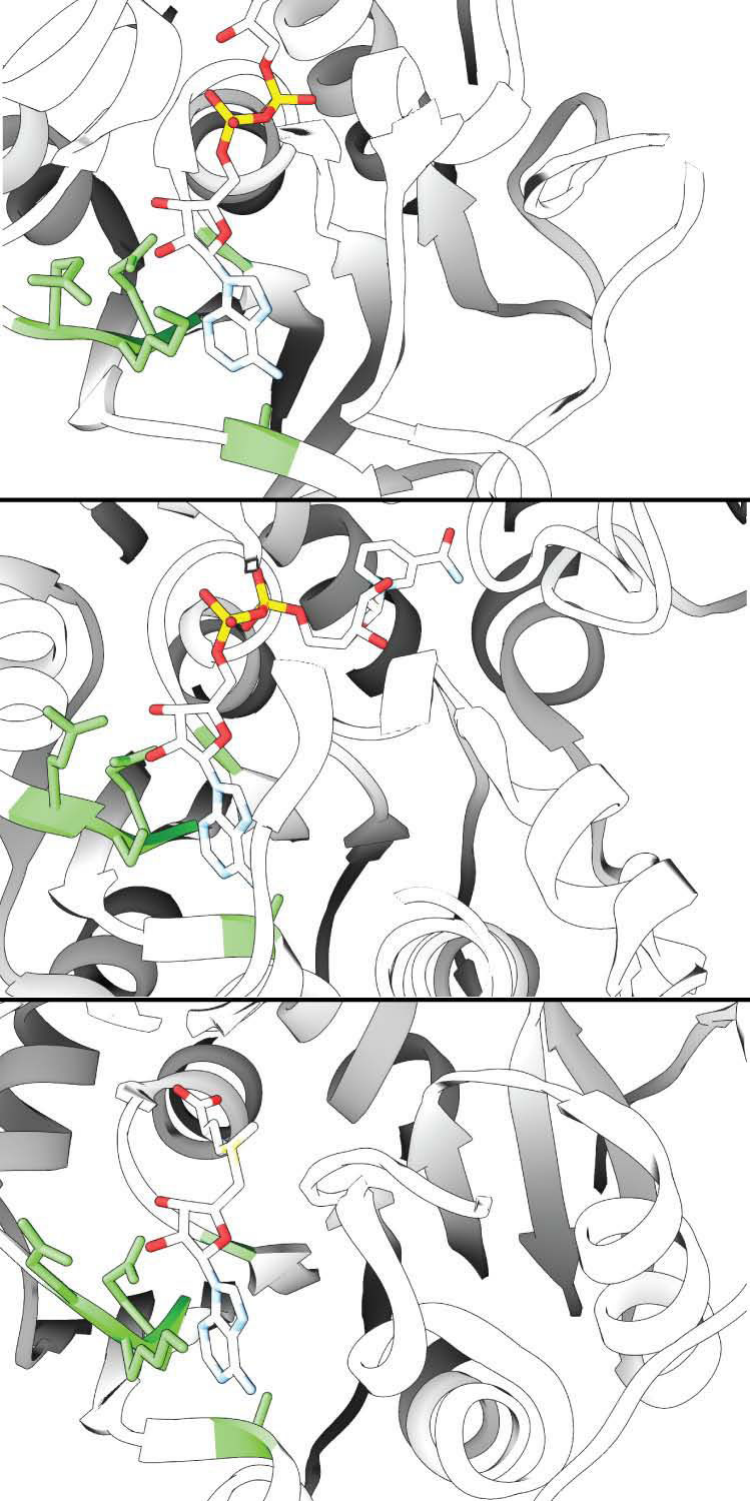
**Matches to adenine-containing ligand binding sites**. Several high-confidence matches identified by the [PDB:1ZQ9]-based motif were identified and subsequently confirmed by the presence of an adenine-containing ligand in the matched site. The matched residues are colored green with the bound ligand colored by atom type. From top to bottom the matches shown above correspond to [PDB:2YQU], [PDB:1W1U], and [PDB:2E58].

Using LabelHash to match the [PDB:1ZQ9]-based motif versus the PDB reveals many additional matches with low *p*-values to binding sites for adenine-containing molecules including FAD, NAD, and SAM. Although LabelHash does not take into account the presence, position, or absence of a bound ligand at any point in the matching process, many of the identified matches could be confirmed as binding sites because of a bound adenine-containing ligand. Matches to lipoamide dehydrogenase [PDB:2YQU], urocanase [PDB:1W1U], and MnmC2 [PDB:2E58] were identified with *p*-values less than 0.0001, 0.0005, and 0.0006, respectively. Each match identified is shown in Figure 7.

#### Catalytic Site Atlas Motifs

In our final experiment we matched a large number of motifs from the Catalytic Site Atlas (CSA) [59] against the nrPDB_95_. The CSA is a manually-curated database of literature-documented functional sites. All experiments were conducted using CSA version 2.2.11 which contains 968 literature-documented sites. The subset of CSA sites used in our experiments includes only literature-documented sites that adhere to the following criteria:

1. The PDB structure corresponding to the site must have a fully qualified EC classification (i.e., 3.4.1.1 is fully qualified but 3.4.1.- or 3.2.- or 3.- are all not); each site will be compared against proteins within the same EC classification to assess sensitivity.

2. The EC class corresponding to the documented site must contain more than 10 structures so that meaningful per-site statistics can be computed for all sites included in the benchmark.

3. The LabelHash motif based upon the CSA site must contain at least one valid reference set (prerequisite for matching; see *Methods *for details on reference set constraints).

4. The site must contain a minimum of 4 non-hetero residues (i.e., residues referencing prosthetic groups, coordinated metal ions, or other ligands do not count towards the 4 residue minimum) because the current LabelHash implementation does not support matching heteroresidues/heteroatoms.

From the available 968 CSA sites, 147 adhered to our criteria. From each of these 147 CSA sites, a LabelHash motif was constructed using the PDB structure that corresponded to each CSA record in every case. Because the CSA does not include allowable amino acid substitutions or mutations for site records, only the single amino acid label that corresponds to the amino acids present in the PDB structure were used in all cases. The motifs analyzed here range in size from 4 to 8 residues, while previous work by Torrance et al. [64] only investigated the performance of CSA motifs ranging from 3-5 residues in size.

To assess the ability of each CSA-based motif to identify functionally equivalent catalytic sites in protein structure, each CSA-based motif was matched against the EC class corresponding to the PDB structure of each site. Structures sharing EC classification with the CSA-based motif are considered positive matches while matches to structures outside of the motif EC class within the nrPDB_95 _were considered negative matches. For the CSA benchmark, the individual chains in both positive and negative protein structures are matched individually. For the 147 CSA-based motifs, 118 unique EC classes are represented spanning all 6 top-level EC classifications (oxidoreductases, transferases, hydrolases, lyases, isomerases, ligases).

Overall, the ability of CSA motifs to identify members of the same EC class (4th-level EC classification) varied wildly. This result is not unexpected due to the fact that per-EC class coverage was not a consideration in the design of the CSA [64]; the aforementioned study by Torrance et al. [64] considered the set of positive structures for each motif to be the "CSA family" of PSI-BLAST identified relatives rather than the full, 4th-level EC class. The analysis performed here widens the study by considering not only those structures with sequence similarity identifiable by PSI-BLAST, but the entire set of structures sharing an EC class with each motif. Highly successful motifs, such as the pyruvate kinase and xylose isomerase motifs based upon structures [PDB:1PKN] and [PDB:2XIS], respectively, correctly identify more than 100 matches to their corresponding EC classes, achieving > 90% EC-class sensitivity with at least 99.8% specificity against the entire nrPDB_95_.

The CSA contains multiple catalytic site definitions for a single EC class in several cases (as can be seen in Additional File 2) and the motifs based on each definition sometimes had large differences in function prediction performance. Consider, for example, two motifs are defined by the CSA for EC:2.1.1.45 (thymidylate synthases): the motif based upon structure [PDB:1LCB] from *Lactobacillus casei *was defined as {198^C^, 219^S^, 221^D^, 257^D^, 259^H^, 60^E^} and matched 192/218 chains with significance resulting in 88.1% sensitivity at a p-value threshold of 0.002, while the motif based upon structure 1TYS from *Escherichia coli *was defined as {146^S^, 166^R^, 169^D^, 58^E^, 94^Y^} matched only 4/218 chains with significance resulting in a drastically lower 1.8% sensitivity at the same *p*-value threshold. Examining the EC:2.1.1.45 matches produced by the poorer performing [PDB:1TYS] motif revealed that a single motif residue (146^S^) was matching a non-cognate serine residue in the majority of EC:2.1.1.45 structures that caused the RMSD of these matches to increase drastically and fall outside of the significance threshold. However, pruning 146^S ^from the [PDB:1TYS] motif increased the sensitivity to 80.3%, matching 175/218 chains in EC:2.1.1.45.

In other cases where multiple motifs were defined by the CSA for a single EC class, each motif was found to match a different subset of functionally related structures. For example, the CSA defines a separate motif for both cellobiohydrolase (CBH) I and II from *Trichoderma reesei *which both belong to EC:3.2.1.91 (cellulose 1,4-*β*-cellobiosidases). Individually, the CBH I and II motifs have a sensitivity of 43.7% and 21.1%, respectively, at *p *= 0.002, but because both motifs match a mutually exclusive set of structures within EC:3.2.1.91, the combination of both motifs identifies over 60% of structures within EC:3.2.1.91. However, combining the results from multiple motifs by taking the union of all positive matches will result in a decrease in overall specificity due to lack of multiple testing correction. In recent work [65], we have developed a technique call Motif Ensemble Statistical Hypothesis testing that allows multiple (potentially overlapping) motifs to be statistically combined into a single function prediction test.

Many of the CSA motifs included in the benchmark could be further optimized to increase function prediction sensitivity and specificity. The previously developed Geometric Sieving (GS) method for motif refinement [34] identifies subsets of motif residues within a larger motif that preserve the overall specificity of the larger motif while increasing sensitivity by removing motif residues that do not contribute to specificity. Also in previous work [34], evolutionary methods based upon Multiple Sequence Alignment (MSA) and phylogenetic tree construction, such as Evolutionary Trace [45] and ConSurf [66], have been used to automatically define motifs and identify alternate residue labels in order to represent residue substitutions present in a MSA. Therefore, while the CSA motifs examined in this benchmark serve as an objective set of expert-defined catalytic site definitions, many motifs could be improved further by applying our previously investigated optimization techniques.

#### Performance and Scalability

**Figure 8:**
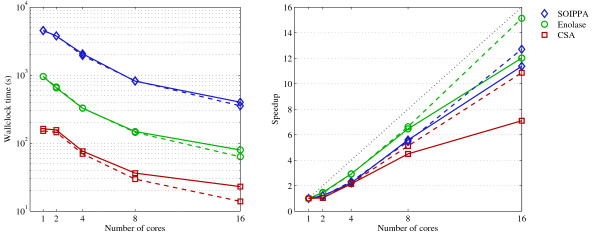
**Performance**. Average wallclock time (**left**) and speedup (**right**) for matching the different categories of motifs against the entire nrPDB_95_. The solid lines indicate performance using a shared Panasas filesystem, while the dashed lines indicate performance with solid-state drives.

Figure 8 shows the average performance of matching all motifs mentioned above against *all *21,745 targets in the nrPDB_95_. The main observation in the left graph is that most motifs can be matched against the entire nrPDB_95 _in a few seconds up to a few minutes. To the best of our knowledge no other general purpose substructure matching algorithm implementation comes even close to such performance. The right graph shows the speedup. The speedup for *n *cores is simply $\frac{T_{1}}{T_{n}}$, where *T_i _*is the wallclock time for *i *cores. The ideal, linear speedup is shown with a black dotted line. Benchmark results for up to 128 cores are shown in Additional File 3.

There were 4 motifs used in the comparison with SOIPPA, 14 motifs in the Enolase Superfamily study, and 147 Catalytic Site Atlas motifs. In general, wallclock time can be reduced significantly by using more cores. The motifs that take more time to match tend to be larger and have more alternate residue labels. For small, simple motifs (3-4 residues without alternate labels) the wallclock time with a single core is already very small and there is not much room for improvement by adding more cores. In this case the runtime is dominated by file access and communication between the cores, with only minimal time needed for computation. The difference between the solid and dashed lines show the performance increase when switching from a shared Panasas filesystem to a local solid-state drive. This difference confirms that much of the time is spent simply reading data. In other words, for simple motifs there is limited room for performance improvement by modifying the match augmentation algorithm. For motifs that take longer to match (the SOIPPA and Enolase superfamily motifs) we see that an almost linear speedup can be expected.

## Conclusions

We have presented LabelHash, a novel algorithm for matching structural motifs. It quickly matches a motif consisting of residue positions, and possible residue types to all structures in the PDB (or any subset thereof). We have shown that LabelHash often achieves very high sensitivity and specificity. The statistical significance of matches is computed using a non-parametric model. Typically, the number of false positive matches is much smaller than the number of true positive matches, despite the large number of targets in our background database. This greatly speeds up the analysis of match results. Our algorithm uses only a small number of parameters whose meaning is easy to understand and which usually do not need to be changed from their default values. Matching results are easily visualized through a plugin for Chimera, a molecular modeling program.

Extensibility was an important factor in the design of the LabelHash implementation. Our program is easily extended to incorporate additional constraints or use even conceptually different types of motifs. For instance, matching based on physicochemical pseudo-centers [22,46] could easily be incorporated, and we plan to offer this functionality in the future. Input and output are all in XML format (except for the LabelHash table itself), which enables easy integration with other tools or web services. As we demonstrated in the study of the enolase superfamily, LabelHash can be part of a multi-stage matching pipeline, where matches produced by LabelHash can be given to the next program, which can apply additional constraints to eliminate more false positives. As long as the set of matches produced by LabelHash include all functional homologs, this is a viable strategy. Of course, the output of LabelHash can also easily be passed on to any clustering algorithm or a visualization front-end.

Our matching algorithm has the capability to keep partial matches and multiple matches per target. This makes the statistical analysis significantly more complicated. Currently, we just disable the *p*-value computation when either option is selected, but we plan to investigate the modeling of the statistical distribution of these matches.

## Methods

### Data sets

For most of our tests we used one LabelHash table for the entire Protein Database (PDB), based on a snapshot taken at March 25, 2009. Each chain was inserted separately in the LabelHash file. This resulted in roughly 138,000 targets. Molecular surfaces (used to compute each residue's depth) were computed with the MSMS software [67]. We chose to use reference sets of size 3. The following parameter values were used for the reference sets: *d*_maxmindist _= 16 Å, *d*_diameter _= 25 Å, *d*_maxmindepth _= 1:6 Å, and *d*_maxdepth _= 3:1 Å. The same parameters were used in all experiments. These values were chosen such that motifs generally contained at least one reference set of size 3. They are very generous in the sense that most motifs we have used for evaluating the LabelHash algorithm contain many reference sets. If reference sets of more than 3 residues are used, the values of the distance parameters need to be increased to guarantee that each motif contains at least one reference set. The advantage of larger reference sets is that we instantly match a larger part of a motif. However, increasing these values also quickly increases the number of reference sets in the targets. So the number of reference sets to perform match augmentation on will also quickly increase. Finally, the storage required for the hash tables grows rapidly with reference set size. After the preprocessing phase the total size of the LabelHash table for the entire PDB was 65 GB, while with reference sets of size 4 (and the same parameters) the table would grow to approximately 5 TB (estimated based on a table constructed for a subset of the PDB).

### Benchmarking

The computational experiments were carried out on a Linux cluster where each node has two quad core 2.4 GHz Intel Xeon (Nehalem) CPUs with 8 MB cache. Each node has 12 GB of memory per node shared by all cores on the node. So for the results in Figure 8 only 2 nodes were used, while for the results in Additional File 3 up to 128/8 = 16 nodes were used. The 65 GB table did not fit on our solid-state drives, so a separate table for just the nrPDB_95 _(21,745 targets, 9.7 GB) was used in the benchmarking tests.

## Authors' contributions

MM and LEK designed the LabelHash algorithm. All authors collectively conceived and designed the experiments and analyzed the resulting data. DHB and MM contributed computational analysis tools/software and performed experiments. All authors wrote the paper, and read and approved the final manuscript.

## Supplementary Material

Additional File 1 **Enolase Superfamily (ES) motifs based on different structures**. The ES motif was defined using different PDB structures as templates and although the amino acid labels are identical across all motifs, the 3 D coordinates of each motif point vary according to the structure on which a motif was based [61]. This causes significant variation in the ability of the motifs to accurately classify ESdb structures. Click here for file

Additional File 2 **Matching results for CSA motifs**. Several motifs obtained from the Catalytic Site Atlas (CSA) were matched against the corresponding protein family and the nrPDB_95_. From the resulting matches the Area Under the Curve (AUC) for the Receiver Operating Characteristic (ROC) curve and Precision-Recall (PR) curve were computed. A ROC curve plots sensitivity $\left(\frac{TP}{TP+FN}\right)$ as a function of the false positive rate $\left(\frac{FP}{FP+TN}\right)$, while a PR curve plots precision $\left(\frac{TP}{TP+FP}\right)$ as a function of recall (which is equivalent to sensitivity). The sensitivity and specificity at a *p*-value threshold of 0.001 were also computed. Click here for file

Additional File 3 **LabelHash parallel performance**. Average wallclock time (**left**) and speedup (**right**) for matching different motifs against the nrPDB_95 _on a high-performance computing cluster. For the motifs used in the SOIPPA comparison and the Enolase Superfamily study substantial (but diminishing) improvements can be obtained by increasing the number of cores. These motifs are larger and have more alternate labels than the CSA. Click here for file
