# Supplementary material for: The LabelHash algorithm for substructure matching
Source: BMC Bioinformatics. 2010 Nov 11;11:555. doi: 10.1186/1471-2105-11-555 (PMC2996407; doi:10.1186/1471-2105-11-555)
Supplement: Additional File 1 — Enolase Superfamily (ES) motifs based on different structures. The ES motif was defined using different PDB structures as templates and although the amino acid labels are identical across all motifs, the 3 D coordinates of each motif point vary according to the structure on which a motif was based [61]. This causes significant variation in the ability of the motifs to accurately classify ESdb structures. [file 1471-2105-11-555-S1.PDF]

Additional File 1 for:
M. Moll, D.H. Bryant, L.E. Kavraki, The LabelHash Algorithm for Substructure Matching, *BMC Bioinformatics*, 2010.

Enolase Superfamily (ES) motifs based on different structures

The ES motif was defined using different PDB structures as templates and although the amino acid labels are identical across all motifs, the 3D coordinates of each motif point vary according to the structure on which a motif was based [1]. This causes significant variation in the ability of the motifs to accurately classify ESdb structures. Below are the enlarged results for the 2MNR-based motif and the following page contains the corresponding results for the remaining structures.

[1] Meng EC, Polacco BJ, Babbitt PC: **Superfamily active site templates.** *Proteins* 2004, **55**(4):962–976.

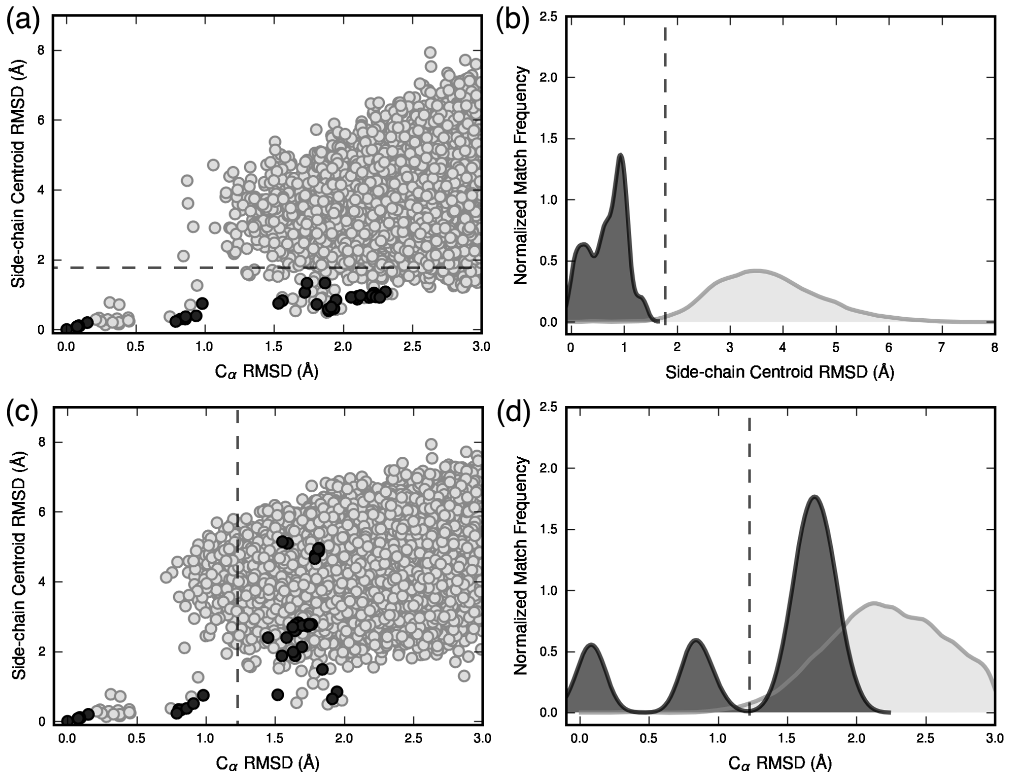

Figure 1: Dark gray denotes matches to structures in the ESdb as defined by Meng et al. [1] while light gray denotes matches to structures in the nrPDB_{95}. The dashed line in each plot corresponds to the *p*-value threshold at $\alpha = 0.01$. The *p*-value threshold is relative to the distribution of side-chain RMSD in **(a)** and **(b)** while relative to C_α RMSD in **(c)** and **(d)**. **(a):** Selecting the "best" match for each individual structure as the match with minimum side-chain RMSD results in a set of ESdb matches that are largely distinguished from the bulk of nrPDB_{95} matches. **(b):** The clear separation of the distributions of ESdb and nrPDB_{95} matches identified using minimum side-chain RMSD illustrates the high-specificity of the 2MNR-based motif. **(c):** Selecting minimum C_α RMSD matches to the ES motif as the "best" matches results in decreased sensitivity at the same $\alpha = 0.01$ threshold due to the high C_α RMSD among ES member substructures. **(d):** Examining the distributions of matches based upon C_α RMSD demonstrates the inseparability of enolase matches from the majority of nrPDB_{95} structures if minimum C_α RMSD is used alone for match selection.

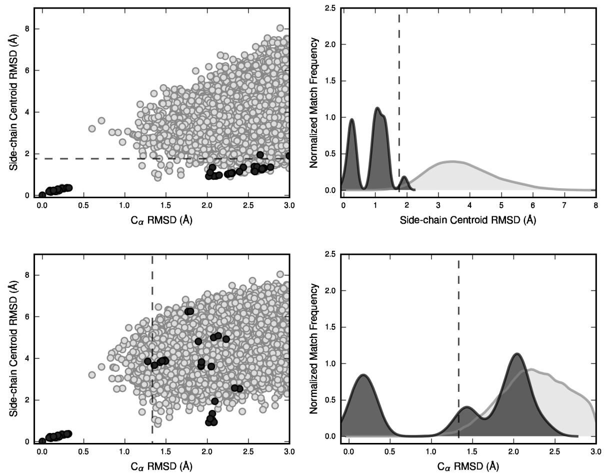

(a) Motif based on 1EBH

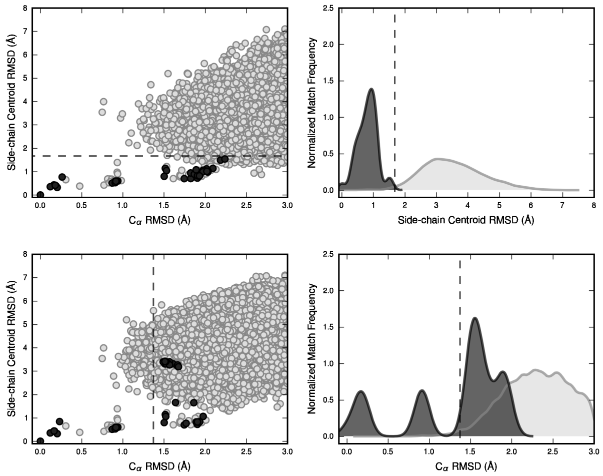

(b) Motif based on 1EC7

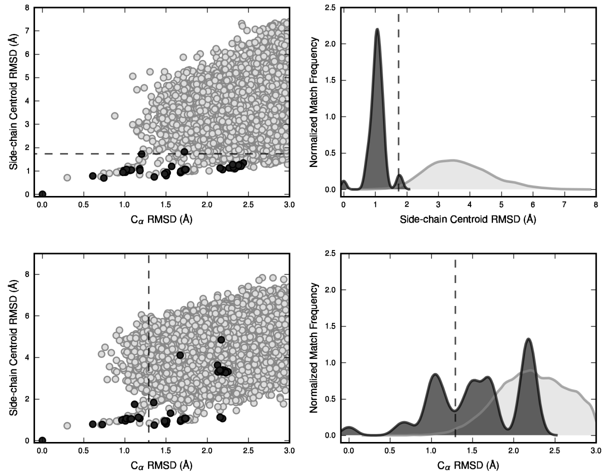

(c) Motif based on 1FHU

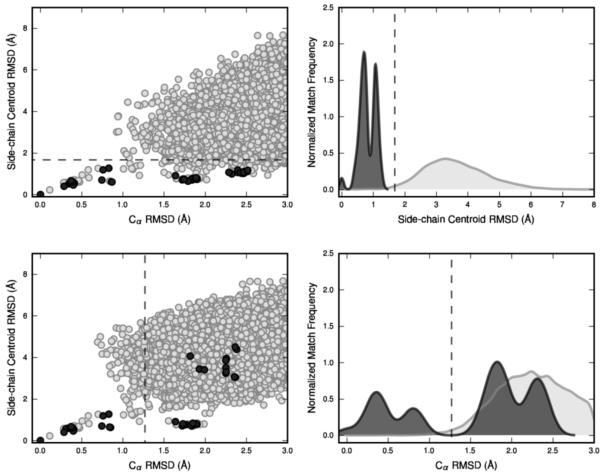

(d) Motif based on 1JPM

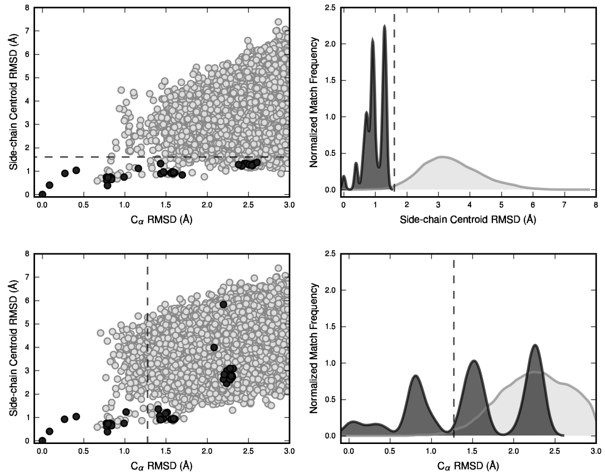

(e) Motif based on 1KKO

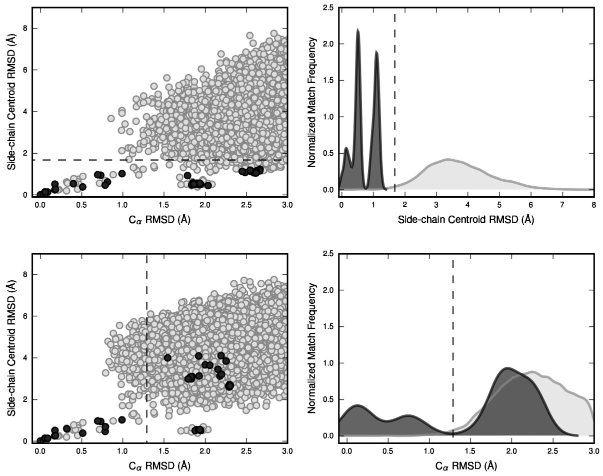

(f) Motif based on 1MUC

Figure 2: The ES motif based on 1EBH has difficulty separating more distant members of the ES from the bulk of matches to unrelated $nrPDB_{95}$ structures, while the 1MUC more accurately distinguishes distant members of the ES (in terms of structural deviation), indicating that 1MUC serves as a better consensus structure for the ESdb.
