# Supplementary material for: The LabelHash algorithm for substructure matching
Source: BMC Bioinformatics. 2010 Nov 11;11:555. doi: 10.1186/1471-2105-11-555 (PMC2996407; doi:10.1186/1471-2105-11-555)
Supplement: Additional File 2 — Matching results for CSA motifs. Several motifs obtained from the Catalytic Site Atlas (CSA) were matched against the corresponding protein family and the nrPDB95. From the resulting matches the Area Under the Curve (AUC) for the Receiver Operating Characteristic (ROC) curve and Precision-Recall (PR) curve were computed. A ROC curve plots sensitivity (TPTP+FN) as a function of the false positive rate (FPFP+TN), while a PR curve plots precision (TPTP+FP) as a function of recall (which is equivalent to sensitivity). The sensitivity and specificity at a p-value threshold of 0.001 were also computed. [file 1471-2105-11-555-S2.PDF]

Additional File 2 for:
M. Moll, D.H. Bryant, L.E. Kavraki, The LabelHash Algorithm for Substructure Matching, *BMC Bioinformatics*, 2010.

Matching results for CSA motifs

Several motifs obtained from the Catalytic Site Atlas (CSA) were matched against the corresponding protein family and the $nrPDB_{95}$. From the resulting matches the Area Under the Curve (AUC) for the Receiver Operating Characteristic (ROC) curve and Precision-Recall (PR) curve were computed. A ROC curve plots sensitivity ($\frac{TP}{TP+FN}$) as a function of the false positive rate ($\frac{FP}{FP+TN}$), while a PR curve plots precision ($\frac{TP}{TP+FP}$) as a function of recall (which is equivalent to sensitivity). The sensitivity and specificity at a p-value threshold of 0.001 were also computed.

Protein family size For both the positive examples (chains belonging to the given EC class) and negative examples (chains within the $nrPDB_{95}$ but not within the EC class), the total number of examples in each case is given by the number of *possible* matches according to label existence criteria. This number is shown in parentheses in the first column. A possible match is one where, within the entirety of all residues constituting a single chain, one or more sets of atoms that share label compatibility with the motif exist. Chains from both the positive and negative sets which do not contain a possible match are excluded from the set of structures matched against *a priori*; this set of excluded structures will vary based upon the given motif and alternate amino acid labels present. For example, if a motif consisting of 5 tryptophan residues is matched against a chain that contains only 4 tryptophan residues, then no match, regardless of the LRMSD value, is possible and thus this chain is excluded. However, any chain that contains 5 or more tryptophan residues would contain a possible match in this case.

Domain of the ROC and PR curves LabelHash limits the maximum LRMSD of matches returned during matching to a user specified limit (the ε distance cutoff). The matches with an LRMSD beyond the distance cutoff are, however, accounted for statistically by using a pointweight at $+\infty$ as discussed in the text. Therefore, the domain of the ROC and PR curves is $[0, 1-pointweight)$ and the curves are only plotted within this domain.

Calculation of AUC values In order to calculate AUC_{ROC} and AUC_{PR} values for each motif over the full interval $[0,1]$, the integral is interpolated linearly between $[1-pointweight, 1]$. Therefore, for a motif with $AUC_{ROC} = 1.0$ the corresponding AUC_{PR} may be calculated to be less than 1.0 due to the linear interpolation of the AUC values within the undefined point weight region $[1-pointweight, 1]$.

Matching results for CSA motifs

Protein family (#)	Motif	AUC_{ROC}	AUC_{PR}	Sensitivity (#TP)	Specificity (#FP)
1.1.1.35 (50)	[PDB:2HDH] 137^{S}, 158^{H}, 170^{E}, 208^{N}	0.75	0.31	38.0% (19)	99.9% (28)
1.1.1.44 (18)	[PDB:2PGD] 130^{G}, 183^{K}, 187^{N}, 190^{E}	1.00	0.33	100.0% (18)	99.9% (27)
1.1.2.3 (22)	[PDB:1FCB] 143^{Y}, 254^{Y}, 282^{D}, 373^{H}, 376^{R}	0.99	0.25	90.9% (20)	99.8% (26)
1.1.3.9 (12)	[PDB:1GOG] 228^{C}, 272^{Y}, 290^{W}, 495^{Y}	0.85	0.14	66.7% (8)	99.8% (23)

Matching results for CSA motifs

Protein family (#)		Motif	AUC_{ROC}	AUC_{PR}	Sensitivity (#TP)	Specificity (#FP)
1.11.1.10 (41)	[PDB:1A8S]	95^{W}, 224^{S}, 28^{T}, 94^{D}, 253^{H}	0.10	0.00	0.0% (0)	99.8% (27)
1.11.1.10 (41)	[PDB:1QI9]	486^{K}, 341^{H}, 418^{H}, 411^{H}	0.68	0.06	19.5% (8)	99.9% (18)
1.14.13.39 (284)	[PDB:3NOS]	356^{C}, 187^{R}, 184^{W}, 361^{E}	0.99	0.96	96.1% (273)	99.9% (11)
1.14.99.3 (113)	[PDB:1DVE]	25^{H}, 143^{Y}, 140^{T}, 136^{R}, 135^{G}, 139^{D}, 58^{G}	0.84	0.55	54.9% (62)	99.9% (26)
1.17.4.1 (112)	[PDB:3R1R]	437^{C}, 441^{N}, 439^{C}, 225^{E}, 462^{C}	0.74	0.26	42.9% (48)	99.9% (13)
1.17.4.1 (133)	[PDB:1QFN]	8^{R}, 13^{G}, 72^{Y}, 18^{K}, 10^{Y}	0.66	0.02	0.8% (1)	99.8% (32)
1.18.1.2 (102)	[PDB:1E6E]	55^{H}, 159^{D}, 377^{I}, 376^{T}	0.73	0.04	3.9% (4)	99.9% (24)
1.2.1.2 (27)	[PDB:1KQF]	800^{W}, 803^{S}, 805^{W}, 806^{T}, 807^{G}, 808^{N}, 809^{D}, 810^{I}	0.50	0.06	7.4% (2)	99.8% (21)
1.2.1.2 (27)	[PDB:2NAC]	313^{N}, 146^{R}, 284^{Q}, 332^{H}	0.06	0.00	0.0% (0)	99.8% (28)
1.2.1.3 (159)	[PDB:1O04]	192^{K}, 268^{E}, 302^{S}, 399^{E}	0.66	0.09	10.1% (16)	99.8% (31)
1.3.3.1 (50)	[PDB:1D3G]	149^{F}, 215^{S}, 218^{T}, 255^{K}	0.87	0.19	24.0% (12)	99.9% (17)
1.3.99.1 (97)	[PDB:1D4C]	364^{H}, 401^{R}, 503^{H}, 544^{R}	0.66	0.15	24.7% (24)	99.9% (24)
1.4.1.1 (40)	[PDB:1PJB]	117^{K}, 269^{H}, 74^{E}, 95^{D}	0.85	0.02	5.0% (2)	99.8% (35)
1.5.1.3 (205)	[PDB:1VIE]	32^{K}, 67^{Q}, 68^{I}, 69^{Y}	0.50	0.02	2.9% (6)	99.8% (35)
1.5.1.3 (207)	[PDB:1RA2]	20^{I}, 27^{M}, 28^{D}, 31^{L}, 5^{F}, 54^{L}, 94^{I}	0.70	0.25	25.6% (53)	99.8% (27)
1.6.4.2 (25)	[PDB:1GET]	177^{C}, 181^{C}, 42^{K}, 47^{Y}, 50^{E}	0.95	0.30	88.0% (22)	99.8% (25)
1.8.1.2 (16)	[PDB:1AOP]	153^{R}, 215^{R}, 217^{K}, 483^{K}, 83^{C}	0.90	0.19	75.0% (12)	99.8% (25)
1.9.3.1 (229)	[PDB:1EHK]	233^{H}, 237^{Y}, 384^{H}, 385^{F}, 386^{H}, 449^{R}, 450^{R}	0.59	0.22	20.1% (46)	99.9% (19)
2.1.1.45 (218)	[PDB:1LCB]	198^{E}, 219^{C}, 221^{S}, 257^{D}, 259^{D}, 60^{H}	0.98	0.90	88.1% (192)	99.8% (24)
2.1.1.45 (218)	[PDB:1TYS]	146^{E}, 166^{Y}, 169^{S}, 58^{R}, 94^{D}	0.85	0.05	1.8% (4)	99.8% (36)
2.1.1.63 (14)	[PDB:1EH6]	137^{N}, 145^{C}, 146^{H}, 172^{E}	0.82	0.57	71.4% (10)	99.9% (22)
2.1.2.2 (48)	[PDB:1C2T]	106^{N}, 108^{H}, 135^{S}, 144^{D}	0.88	0.17	20.8% (10)	99.8% (37)
2.1.2.2 (48)	[PDB:1GRC]	106^{N}, 108^{H}, 135^{S}, 144^{D}	0.79	0.03	14.6% (7)	99.8% (32)
2.1.3.2 (235)	[PDB:1AT1]	105^{R}, 134^{T}, 54^{R}, 55^{H}	0.82	0.52	53.2% (125)	99.9% (25)
2.1.3.3 (49)	[PDB:1AKM]	106^{R}, 133^{H}, 136^{Q}, 231^{D}, 273^{C}, 319^{R}	1.00	0.89	93.9% (46)	99.9% (19)
2.3.1.41 (173)	[PDB:1DD8]	163^{C}, 298^{H}, 328^{K}, 333^{H}, 390^{F}, 392^{F}	0.71	0.42	42.8% (74)	99.9% (18)
2.3.1.41 (173)	[PDB:1KAS]	163^{C}, 303^{H}, 340^{H}, 400^{F}	0.78	0.52	52.6% (91)	99.9% (17)
2.3.1.74 (41)	[PDB:1CGK]	164^{C}, 215^{F}, 303^{H}, 336^{N}	0.65	0.08	29.3% (12)	99.8% (26)
2.3.2.13 (43)	[PDB:1IU4]	255^{C}, 269^{D}, 272^{K}, 274^{W}, 64^{H}	0.62	0.03	9.3% (4)	99.8% (22)
2.4.1.1 (189)	[PDB:1GPA]	568^{K}, 569^{R}, 574^{K}, 676^{T}	0.81	0.18	33.9% (64)	99.8% (31)
2.4.1.19 (61)	[PDB:1CDG]	227^{R}, 229^{D}, 257^{E}, 327^{H}, 328^{D}	0.97	0.71	80.3% (49)	99.8% (34)

Matching results for CSA motifs

Protein family (#)	Motif	AUC_{ROC}	AUC_{PR}	Sensitivity (#TP)	Specificity (#FP)
2.4.2.6 (29)	[PDB:1F8X] 207^{Y}, 272^{D}, 292^{D}, 298^{E}	0.82	0.42	55.2% (16)	99.9% (27)
2.4.2.8 (76)	[PDB:1BZY] 133^{E}, 134^{D}, 137^{D}, 165^{K}, 169^{R}	0.91	0.45	55.3% (42)	99.8% (33)
2.5.1.19 (45)	[PDB:1G6T] 22^{K}, 313^{D}, 341^{E}, 385^{H}, 411^{K}	0.69	0.09	35.6% (16)	99.8% (32)
2.5.1.31 (38)	[PDB:1F75] 197^{R}, 203^{R}, 33^{R}, 42^{R}	0.12	0.00	0.0% (0)	99.8% (30)
2.5.1.6 (28)	[PDB:1FUG] 14^{H}, 165^{K}, 244^{R}, 245^{K}	1.00	0.88	100.0% (28)	99.9% (26)
2.5.1.7 (45)	[PDB:1UAE] 115^{N}, 23^{C}, 305^{D}, 397^{R}	0.05	0.00	0.0% (0)	99.8% (25)
2.5.1.9 (130)	[PDB:1I8D] 102^{F}, 2^{S}, 41^{C}, 48^{M}, 64^{H}	0.46	0.03	2.3% (3)	99.9% (16)
2.5.1.9 (185)	[PDB:1KZL] 146^{S}, 185^{C}, 41^{H}, 48^{S}, 97^{D}	0.29	0.01	2.2% (4)	99.8% (25)
2.6.1.16 (37)	[PDB:1JXA] 481^{E}, 485^{K}, 488^{E}, 603^{K}	0.72	0.28	35.1% (13)	99.9% (27)
2.6.1.16 (37)	[PDB:1MOQ] 481^{E}, 485^{K}, 488^{E}, 603^{K}	0.73	0.11	35.1% (13)	99.9% (23)
2.7.1.21 (101)	[PDB:1KIM] 163^{G}, 222^{E}, 59^{R}, 83^{R}	0.68	0.06	16.8% (17)	99.8% (37)
2.7.1.40 (119)	[PDB:1PKN] 119^{R}, 269^{R}, 327^{K}, 361^{T}, 363^{S}, 72^{E}	1.00	0.92	91.6% (109)	99.8% (31)
2.7.1.69 (55)	[PDB:1GPR] 66^{T}, 68^{H}, 83^{H}, 85^{G}	0.56	0.04	16.4% (9)	99.9% (17)
2.7.4.3 (73)	[PDB:1ZIO] 127^{K}, 13^{R}, 160^{R}, 162^{D}, 163^{D}, 171^{R}	0.15	0.00	0.0% (0)	99.9% (24)
2.8.1.1 (16)	[PDB:1E0C] 231^{Q}, 232^{T}, 233^{H}, 234^{H}, 235^{R}	0.60	0.14	18.8% (3)	99.8% (25)
2.8.1.1 (16)	[PDB:1RHS] 186^{R}, 248^{R}, 249^{K}, 250^{G}, 251^{V}, 252^{T}	0.75	0.31	43.8% (7)	99.8% (38)
3.1.1.1 (115)	[PDB:1AUO] 114^{L}, 115^{S}, 168^{Q}, 199^{D}, 23^{H}	0.64	0.07	8.7% (10)	99.8% (31)
3.1.1.1 (115)	[PDB:1CI8] 181^{S}, 348^{K}, 351^{Y}, 75^{W}, 78^{V}	0.55	0.03	3.5% (4)	99.8% (27)
3.1.1.1 (115)	[PDB:1L7Q] 117^{Y}, 118^{A}, 166^{Y}, 259^{W}, 287^{D}, 44^{H}	0.73	0.02	0.9% (1)	99.8% (26)
3.1.1.3 (164)	[PDB:1R4Z] 12^{I}, 133^{S}, 156^{M}, 77^{D}, 78^{H}	0.61	0.10	20.7% (34)	99.8% (24)
3.1.1.3 (192)	[PDB:2LIP] 17^{L}, 264^{S}, 286^{Q}, 87^{D}, 88^{H}	0.79	0.19	16.1% (31)	99.8% (32)
3.1.1.4 (347)	[PDB:1CJY] 197^{G}, 198^{G}, 228^{S}, 549^{D}	0.45	0.01	0.6% (2)	99.8% (32)
3.1.1.47 (31)	[PDB:1BWP] 104^{S}, 192^{G}, 195^{N}, 47^{D}, 74^{H}	0.07	0.00	0.0% (0)	99.8% (33)
3.1.11.2 (23)	[PDB:1AKO] 151^{N}, 153^{D}, 229^{N}, 259^{D}, 7^{H}	0.29	0.01	13.0% (3)	99.9% (26)
3.1.2.15 (51)	[PDB:1CMX] 484^{Q}, 490^{C}, 566^{H}, 581^{D}	0.77	0.07	7.8% (4)	99.8% (25)
3.1.2.15 (51)	[PDB:1NBF] 218^{N}, 223^{C}, 464^{H}, 481^{D}	0.74	0.03	9.8% (5)	99.8% (23)
3.1.2.15 (51)	[PDB:1UCH] 169^{Q}, 184^{C}, 89^{H}, 95^{D}	0.77	0.04	5.9% (3)	99.8% (23)
3.1.21.1 (52)	[PDB:1FR2] 100^{R}, 102^{E}, 103^{H}, 5^{H}	0.69	0.07	26.9% (14)	99.9% (21)
3.1.27.5 (315)	[PDB:1RBN] 119^{H}, 12^{K}, 120^{H}, 41^{F}	0.90	0.74	72.1% (227)	99.9% (17)
3.1.3.16 (107)	[PDB:1S95] 274^{D}, 275^{R}, 303^{N}, 304^{H}, 400^{R}, 427^{H}	0.69	0.16	35.5% (38)	99.9% (21)
3.1.3.2 (131)	[PDB:1D2T] 150^{H}, 183^{R}, 189^{H}, 193^{D}	0.62	0.13	13.7% (18)	99.8% (25)

Matching results for CSA motifs

Protein family (#)	Motif	AUC_{ROC}	AUC_{PR}	Sensitivity (#TP)	Specificity (#FP)
3.1.3.2 (131)	[PDB:1RPT] 11^{R}, 12^{H}, 15^{R}, 257^{R}, 258^{H}, 79^{D}	0.25	0.00	0.0% (0)	99.8% (24)
3.1.3.2 (65)	[PDB:1PNT] 12^{C}, 129^{C}, 17^{R}, 18^{D}	0.73	0.10	12.3% (8)	99.9% (17)
3.1.3.3 (25)	[PDB:1L7N] 100^{D}, 11^{F}, 12^{D}, 13^{G}, 144^{K}, 171^{D}	0.79	0.16	20.0% (5)	99.8% (33)
3.1.3.48 (328)	[PDB:1BZC] 181^{D}, 215^{C}, 221^{R}, 222^{S}	0.77	0.18	27.4% (90)	99.9% (20)
3.1.3.48 (328)	[PDB:1PA9] 194^{D}, 241^{C}, 247^{R}, 248^{T}	0.76	0.08	8.5% (28)	99.9% (20)
3.1.3.48 (328)	[PDB:1YTW] 290^{E}, 356^{D}, 402^{H}, 403^{C}, 409^{R}, 410^{T}	0.85	0.46	16.8% (55)	99.9% (13)
3.1.3.48 (346)	[PDB:1D1Q] 13^{A}, 132^{R}, 19^{S}, 20^{D}	0.66	0.04	2.0% (7)	99.8% (31)
3.1.3.5 (60)	[PDB:1USH] 116^{N}, 117^{H}, 120^{D}, 375^{R}, 379^{R}, 410^{R}	0.50	0.01	5.0% (3)	99.8% (27)
3.1.4.10 (11)	[PDB:1PTD] 274^{H}, 32^{R}, 69^{H}, 82^{D}	0.79	0.40	54.5% (6)	99.9% (23)
3.1.4.10 (11)	[PDB:2PLC] 278^{H}, 45^{D}, 46^{R}, 84^{H}, 93^{D}	0.88	0.45	63.6% (7)	99.8% (26)
3.2.1.113 (19)	[PDB:1DL2] 132^{E}, 136^{R}, 275^{D}, 435^{E}	1.00	0.80	100.0% (19)	99.9% (27)
3.2.1.135 (38)	[PDB:1BVZ] 325^{D}, 354^{E}, 356^{W}, 421^{D}	0.86	0.28	36.8% (14)	99.8% (28)
3.2.1.17 (1276)	[PDB:1OBA] 10^{D}, 182^{D}, 92^{E}, 94^{D}	0.30	0.04	0.5% (6)	99.8% (33)
3.2.1.18 (270)	[PDB:7NN9] 151^{D}, 220^{R}, 277^{E}, 371^{R}, 412^{D}	0.85	0.14	8.1% (22)	99.8% (29)
3.2.1.35 (14)	[PDB:1FCQ] 111^{D}, 113^{E}, 184^{Y}, 227^{Y}, 301^{W}	0.87	0.08	42.9% (6)	99.8% (28)
3.2.1.73 (24)	[PDB:1AQ0] 232^{E}, 280^{E}, 283^{K}, 288^{E}	0.92	0.02	12.5% (3)	99.8% (37)
3.2.1.78 (24)	[PDB:1BQC] 127^{N}, 128^{E}, 196^{H}, 198^{Y}, 225^{E}	0.10	0.00	0.0% (0)	99.8% (29)
3.2.1.8 (226)	[PDB:1XYZ] 645^{E}, 723^{H}, 754^{E}, 756^{D}	0.57	0.19	38.5% (87)	99.9% (17)
3.2.1.91 (71)	[PDB:1CEL] 212^{E}, 214^{D}, 217^{E}, 228^{H}	0.74	0.38	43.7% (31)	99.9% (28)
3.2.1.91 (71)	[PDB:1QK2] 169^{Y}, 174^{R}, 175^{D}, 221^{D}	0.85	0.07	21.1% (15)	99.8% (30)
3.2.1.8 (226)	[PDB:1EXP] 127^{E}, 205^{H}, 233^{E}, 235^{D}	0.57	0.19	38.5% (87)	99.9% (19)
3.2.2.1 (32)	[PDB:1R4F] 10^{D}, 186^{W}, 260^{N}, 83^{A}	0.93	0.02	3.1% (1)	99.8% (25)
3.2.2.23 (17)	[PDB:1K82] 1^{P}, 2^{E}, 258^{K}, 56^{R}	0.95	0.09	41.2% (7)	99.8% (34)
3.2.3.1 (19)	[PDB:1MYR] 187^{R}, 190^{Q}, 328^{S}, 330^{N}, 409^{Y}, 95^{E}	0.99	0.22	89.5% (17)	99.8% (36)
3.3.1.1 (71)	[PDB:1B3R] 130^{H}, 185^{D}, 189^{K}, 190^{D}, 194^{N}, 300^{C}, 54^{H}	1.00	0.91	84.5% (60)	99.8% (20)
3.3.2.3 (24)	[PDB:1EHY] 107^{W}, 108^{D}, 152^{F}, 215^{Y}, 246^{Y}, 275^{D}, 38^{H}	0.99	0.23	25.0% (6)	99.8% (24)
3.4.15.1 (16)	[PDB:1O8A] 353^{H}, 354^{A}, 384^{E}, 513^{H}, 523^{Y}	0.97	0.75	93.8% (15)	99.8% (32)
3.4.19.3 (22)	[PDB:1AUG] 291^{E}, 301^{R}, 354^{C}, 378^{H}	0.94	0.77	86.4% (19)	99.9% (17)
3.4.21.12 (39)	[PDB:1SSX] 102^{H}, 193^{D}, 195^{G}, 214^{S}, 57^{S}	1.00	0.48	100.0% (39)	99.8% (43)
3.4.21.7 (29)	[PDB:1DDJ] 603^{H}, 646^{D}, 737^{C}, 738^{Q}, 739^{G}, 740^{D}, 741^{A}	0.57	0.19	27.6% (8)	99.8% (27)
3.4.21.81 (52)	[PDB:1DS2] 102^{H}, 193^{D}, 195^{G}, 57^{S}	0.55	0.20	50.0% (26)	99.8% (39)

Matching results for CSA motifs

Protein family (#)		Motif	AUC_{ROC}	AUC_{PR}	Sensitivity (#TP)	Specificity (#FP)
3.4.21.92 (180)	[PDB:1TYF]	$122^G, 171^S, 68^M, 97^H, 98^D$	0.92	0.80	81.7% (147)	99.9% (15)
3.4.21.97 (29)	[PDB:1IEC]	$134^H, 157^S, 165^A, 166^R, 63^R$	0.95	0.08	6.9% (2)	99.8% (35)
3.4.21.97 (29)	[PDB:1NKK]	$132^H, 157^S, 165^H, 63^R$	0.91	0.57	69.0% (20)	99.9% (23)
3.4.22.17 (24)	[PDB:1KFU]	$105^Q, 262^C, 286^H, 99^N$	0.31	0.01	8.3% (2)	99.9% (21)
3.4.22.17 (24)	[PDB:1KFX]	$105^Q, 262^C, 286^H, 288^N, 99^W$	0.43	0.01	8.3% (2)	99.8% (22)
3.4.22.27 (37)	[PDB:1GLO]	$164^Q, 184^S, 19^H, 25^N$	0.67	0.03	2.7% (1)	99.8% (31)
3.4.22.28 (20)	[PDB:2BHG]	$163^H, 182^D, 46^A, 84^S$	0.45	0.02	20.0% (4)	99.9% (28)
3.4.22.36 (55)	[PDB:2FQQ]	$237^H, 238^G, 285^A, 286^R$	0.63	0.07	9.1% (5)	99.8% (32)
3.4.23.20 (14)	[PDB:1APT]	$213^D, 216^S, 33^D, 36^T$	1.00	0.73	100.0% (14)	99.8% (36)
3.4.23.22 (22)	[PDB:1EED]	$215^D, 218^S, 32^D, 35^T$	1.00	0.75	100.0% (22)	99.8% (38)
3.4.23.25 (12)	[PDB:2JXR]	$215^D, 218^T, 32^D, 33^T$	1.00	0.17	100.0% (12)	99.8% (31)
3.4.23.6 (20)	[PDB:2APR]	$218^D, 221^S, 35^D, 38^T$	1.00	0.76	100.0% (20)	99.8% (35)
3.5.1.26 (34)	[PDB:1APY]	$183^T, 201^T, 234^T, 235^G$	0.65	0.15	20.6% (7)	99.9% (26)
3.5.1.31 (42)	[PDB:1BS4]	$133^G, 45^Q, 50^L, 91^E$	0.99	0.79	95.2% (40)	99.8% (36)
3.5.1.5 (106)	[PDB:1KRA]	$219^H, 221^D, 320^H, 336^R$	0.57	0.08	9.4% (10)	99.9% (17)
3.5.2.6 (506)	[PDB:1BTL]	$130^S, 166^K, 70^S, 73^E$	0.78	0.35	28.1% (142)	99.9% (22)
3.6.1.34 (90)	[PDB:1EF0]	$1^A, 433^N, 434^T, 454^H, 455^G, 76^I, 78^A, 79^C$	0.56	0.01	2.2% (2)	99.9% (20)
4.1.1.1 (38)	[PDB:1PVD]	$114^D, 115^H, 28^H, 477^E$	0.95	0.42	50.0% (19)	99.8% (27)
4.1.1.23 (137)	[PDB:1EIX]	$44^K, 71^D, 73^K, 76^D$	0.90	0.77	71.5% (98)	99.9% (22)
4.1.1.39 (318)	[PDB:1RBA]	$166^K, 191^K, 192^N, 287^H, 321^H$	0.78	0.15	3.1% (10)	99.8% (29)
4.1.3.7 (41)	[PDB:1AL6]	$244^S, 274^H, 320^H, 375^D$	0.94	0.21	56.1% (23)	99.8% (28)
4.2.1.10 (174)	[PDB:1UQR]	$100^N, 107^R, 11^Y, 18^E, 23^H, 98^R$	0.47	0.01	1.1% (2)	99.8% (29)
4.2.1.11 (64)	[PDB:1ELS]	$168^E, 211^E, 373^H, 396^K$	0.92	0.47	51.6% (33)	99.9% (27)
4.2.1.11 (64)	[PDB:5ENL]	$168^E, 211^E, 345^K, 373^H$	0.83	0.19	51.6% (33)	99.9% (26)
4.2.1.17 (57)	[PDB:1DUB]	$141^A, 144^G, 164^E, 98^E$	0.91	0.60	63.2% (36)	99.9% (27)
4.2.1.20 (152)	[PDB:1A50]	$167^H, 305^K, 86^K, 87^D$	0.74	0.09	9.9% (15)	99.8% (32)
4.2.1.92 (19)	[PDB:1U5U]	$137^T, 193^H, 66^N, 67^Y$	0.80	0.01	10.5% (2)	99.8% (30)
4.2.3.4 (34)	[PDB:1UJN]	$131^K, 210^K, 220^E, 224^R, 228^N, 235^H$	0.98	0.54	64.7% (22)	99.8% (33)
4.2.99.18 (22)	[PDB:1BIX]	$171^Y, 210^D, 283^D, 309^H$	0.87	0.67	81.8% (18)	99.9% (26)
5.1.1.3 (38)	[PDB:1B73]	$178^D, 7^S, 70^C, 8^C$	0.62	0.16	18.4% (7)	99.9% (15)
5.2.1.8 (285)	[PDB:1M9C]	$102^R, 113^F, 122^Q, 55^N, 60^F, 63^L$	0.67	0.28	49.1% (140)	99.9% (13)

Matching results for CSA motifs

Protein family (#)	Motif		AUC_{ROC}	AUC_{PR}	Sensitivity (#TP)	Specificity (#FP)
5.3.1.1 (271)	[PDB:1HTI]	11^{N}, 13^{K}, 165^{H}, 171^{E}, 95^{G}	0.08	0.01	0.0% (0)	99.8% (28)
5.3.1.1 (271)	[PDB:1TPH]	11^{N}, 13^{K}, 165^{H}, 171^{E}, 95^{G}	0.93	0.56	41.7% (113)	99.9% (22)
5.3.1.5 (155)	[PDB:2XIS]	181^{H}, 183^{D}, 54^{E}, 57^{K}	0.99	0.46	94.8% (147)	99.9% (20)
5.3.1.6 (65)	[PDB:1NN4]	10^{D}, 137^{H}, 66^{C}, 9^{R}	0.48	0.12	15.4% (10)	99.8% (24)
5.3.1.9 (97)	[PDB:1DQR]	210^{K}, 216^{E}, 271^{G}, 272^{R}, 357^{E}, 518^{K}	0.96	0.72	74.2% (72)	99.9% (29)
5.3.3.2 (50)	[PDB:1I9A]	1067^{C}, 1087^{E}, 1116^{E}, 1161^{W}	0.85	0.54	58.0% (29)	99.9% (17)
5.3.4.1 (26)	[PDB:1EEJ]	100^{C}, 101^{Y}, 125^{C}, 98^{R}	0.70	0.04	15.4% (4)	99.9% (16)
5.3.4.1 (26)	[PDB:1MEK]	36^{C}, 37^{G}, 38^{H}, 39^{C}	0.83	0.26	57.7% (15)	99.9% (13)
5.4.2.1 (47)	[PDB:1QHF]	181^{H}, 59^{R}, 8^{E}, 86^{H}	0.96	0.76	85.1% (40)	99.9% (21)
5.4.2.8 (23)	[PDB:1P5D]	109^{R}, 118^{H}, 20^{K}, 247^{R}, 329^{H}	0.70	0.22	30.4% (7)	99.8% (28)
5.4.99.5 (72)	[PDB:3CSM]	157^{R}, 16^{R}, 168^{K}, 246^{E}	0.78	0.07	6.9% (5)	99.8% (34)
5.99.1.2 (51)	[PDB:1A41]	167^{K}, 223^{R}, 265^{H}, 274^{Y}	0.81	0.03	9.8% (5)	99.9% (24)
5.99.1.2 (51)	[PDB:1ECL]	111^{E}, 319^{D}, 365^{Y}, 9^{H}	0.73	0.23	25.5% (13)	99.8% (31)
5.99.1.2 (53)	[PDB:1D6M]	328^{E}, 330^{K}, 7^{Y}, 8^{R}	0.87	0.06	15.1% (8)	99.8% (33)
6.1.1.1 (66)	[PDB:2TS1]	230^{K}, 233^{R}, 82^{K}, 86^{K}	0.79	0.12	12.1% (8)	99.8% (31)
6.1.1.11 (24)	[PDB:1SES]	256^{R}, 258^{E}, 261^{S}, 265^{D}, 271^{R}	0.94	0.08	12.5% (3)	99.9% (29)
6.3.2.1 (34)	[PDB:2A86]	160^{M}, 196^{H}, 197^{H}, 198^{K}, 40^{S}, 44^{S}, 47^{R}	1.00	0.87	94.1% (32)	99.9% (19)
6.3.2.4 (33)	[PDB:2DLN]	15^{E}, 150^{S}, 216^{Y}, 255^{R}, 276^{G}	0.80	0.15	21.2% (7)	99.8% (34)
6.3.3.3 (14)	[PDB:1DAE]	11^{T}, 15^{K}, 37^{K}, 41^{S}	0.98	0.62	85.7% (12)	99.9% (28)
