# Supplementary material for: The LabelHash algorithm for substructure matching
Source: BMC Bioinformatics. 2010 Nov 11;11:555. doi: 10.1186/1471-2105-11-555 (PMC2996407; doi:10.1186/1471-2105-11-555)

Additional File 3 for:
M. Moll, D.H. Bryant, L.E. Kavraki, The LabelHash Algorithm for Substructure Matching, *BMC Bioinformatics*, 2010.

LabelHash Parallel Performance

Average wallclock time (**left**) and speedup (**right**) for matching different motifs against the nrPDB$_{95}$ on a high-performance computing cluster. For the motifs used in the SOIPPA comparison and the Enolase Superfamily study substantial (but diminishing) improvements can be obtained by increasing the number of cores. These motifs are larger and have more alternate labels than the CSA motifs.

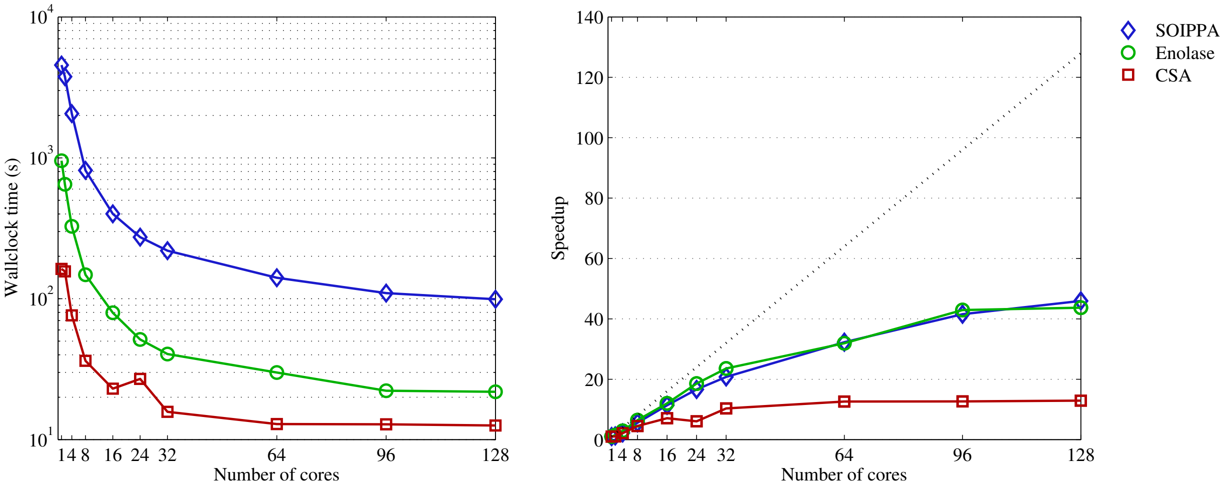

Supplement: Additional File 3 — LabelHash parallel performance. Average wallclock time (left) and speedup (right) for matching different motifs against the nrPDB95 on a high-performance computing cluster. For the motifs used in the SOIPPA comparison and the Enolase Superfamily study substantial (but diminishing) improvements can be obtained by increasing the number of cores. These motifs are larger and have more alternate labels than the CSA. [file 1471-2105-11-555-S3.PDF]
